# Current Practices in CKD-Associated Pruritus: International Nephrologist Survey

**DOI:** 10.1016/j.ekir.2023.04.003

**Published:** 2023-04-07

**Authors:** James O. Burton, Sebastian Walpen, Sandrine Danel, Bernd Schröppel

**Affiliations:** 1 Department of Cardiovascular Sciences, University of Leicester and University Hospitals of Leicester, Leicester, UK; 2 Vifor Pharma Group, Glattbrugg, Switzerland; 3 Section of Nephrology, University Hospital, Ulm, Germany

## Introduction

Chronic kidney disease-associated pruritus (CKD-aP) is common in patients on maintenance dialysis, impacting their quality of life (QoL).^1^, ^2^, ^3^ CKD-aP prevalence may be underestimated and underdiagnosed.^4^ Data from Dialysis Outcomes and Practice Patterns Studies (DOPPS) found that 17% of patients on hemodialysis who were nearly or always bothered by itch did not report symptoms to a health care professional.^2^ Patients reported being unaware of CKD-aP causes and/or accepting CKD-aP as something to live with; lack of concern and/or knowledge regarding CKD-aP by health care professionals; or hesitancy of health care professionals to discuss pruritus, partly because of a previous lack of approved treatments.^4^ Evidence suggests CKD-aP is also undertreated once diagnosed.^2^

The aim of this real-world study was to gain insights from nephrologists in Europe and Australia into current practices, attitudes, and unmet needs regarding CKD-aP diagnosis and treatment.

## Results

### Physician Perception Survey

The survey was completed by 301 nephrologists from Italy (*n* = 58), Germany (*n* = 56), Spain (*n* = 55), UK (*n* = 52), France (*n* = 50), and Australia (*n* = 30) (Supplementary Appendix, Supplementary Table S1) as described in the Supplementary Methods.

Overall, nephrologist-perceived prevalence of CKD-aP was 43.6% (Supplementary Figure S1A). Most nephrologists (79%) used a mild-moderate-severe classification for CKD-aP severity, as opposed to itch/no itch or no classification at all (Supplementary Figure S1B). Most nephrologists classified occasional itching as mild (86%), whereas continuous itching (54%) or itching despite use of topical treatments (70%), dialysis optimization (58%), or antihistamine use (60%) were classified as moderate. Severe itching was classified as itching despite gabapentinoids (77%); visible scratch marks (66%) or abrasions (78%); or impact on sleep (80%), mood (77%), and daily activities (77%) (Supplementary Table S2). Nephrologists estimated that 40% of CKD-aP patients experienced mild itching, 31% experienced moderate itching, and 24% experienced severe itching (Supplementary Figure S1C).

**Figure 1:**
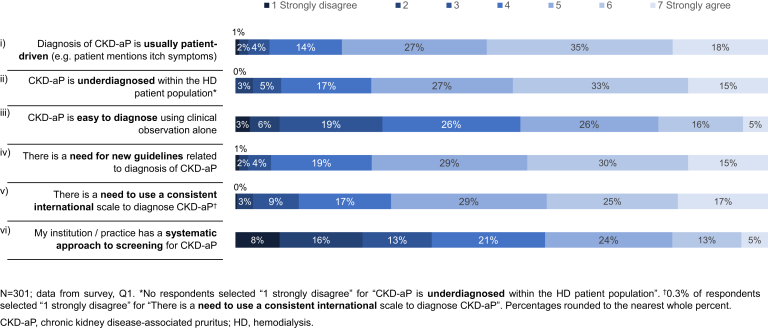
Nephrologists’ level of agreement with statements relating to CKD-aP diagnosis and guidelines.

Overall, 18% of nephrologists strongly agreed and 35% of nephrologists moderately agreed that CKD-aP diagnosis was usually patient-driven; whereas 48% strongly or moderately agreed that CKD-aP was underdiagnosed. In contrast, 21% of respondents strongly or moderately agreed that CKD-aP was easy to diagnose using clinical observation alone, whereas only 18% strongly or moderately agreed with the statement “My institution/practice has a systematic approach to screening for CKD-aP.” A large proportion of nephrologists strongly or moderately agreed that new guidelines (45%) and a consistent international scale (42%) for diagnosing CKD-aP are needed (Figure 1). Only 21% of nephrologists reported using itch scales to determine CKD-aP severity (Supplementary Figure S2A), with the numerical rating scale/visual analog scale most frequently employed (Supplementary Figure S2B).

**Figure 2:**
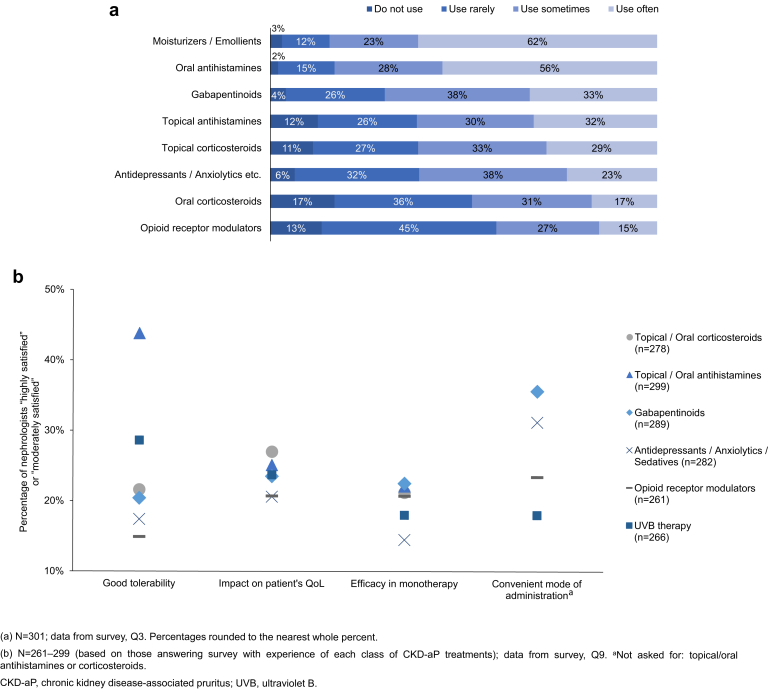
Nephrologists’ perception of CKD-aP treatment: (a) nephrologist awareness and/or usage of CKD-aP treatments; (b) percentage of nephrologists rating “highly satisfied” (score 7) or “moderately satisfied” (score 6) for each CKD-aP treatment.

Frequent use of moisturizers and emollients, antihistamines, and gabapentinoids was noted by 62%, 56%, and 33% of nephrologists, respectively, whereas rare use of opioid receptor modulators and oral corticosteroids was noted by 45% and 36% of respondents, respectively (Figure 2a, and Supplementary Figure S3 for country-specific data). Less than half of the nephrologists were highly satisfied with the tolerability of any of the treatments listed, and less than 30% were highly satisfied with the impact of each medication on patient QoL, or efficacy as a monotherapy (Figure 2b, Supplementary Table S3).

When asked whether “CKD-aP represents a minor concern when considering the broader context of a patient’s CKD,” 7% of nephrologists did not agree at all, whereas 4% strongly agreed (Supplementary Figure S4Ai). Despite this division, the level of agreement with the statement “There is a need for new treatments specifically designed to address CKD-aP” was high, with 26% of respondents strongly agreeing, and 37% moderately agreeing (Supplementary Figure S4Aii). In follow-up questions, nephrologists noted the need for treatments to better reduce the effect of itch, improve patient QoL, and overcome the emotional impact of CKD-aP (Supplementary Figure S4B).

### Patient Record Form Data

Data were captured for 1435 hemodialysis patients with CKD-aP from Italy (*n* = 290), Germany (*n* = 280), Spain (*n* = 275), UK (*n* = 260), France (*n* = 250), and Australia (*n* = 80). Patient record form (PRF) data represented 8% to 19% of the nephrologist-perceived number of center-based hemodialysis patients with CKD-aP seen in the past month (Italy, 8%; Germany, 14%; Spain, 11%; UK, 16%; France, 12%; and Australia, 16%).

Classifications in PRF data were mild (32% of patients), moderate (45%), and severe CKD-aP (20%) (Supplementary Figure S5A). Most patients experienced CKD-aP for 6 months (31%) or 6 months to 1 year (27%), and 5% of patients experienced CKD-aP for over 3 years (Supplementary Figure S5B). Patients reported that the arms, back, and torso were most frequently affected by itch (Supplementary Figure S5C).

Overall, dialysis optimization, moisturizers/emollients, and oral antihistamines were common treatment choices for all 3 lines of therapy. From second-line of therapy onwards, more patients received gabapentinoids, whereas ultraviolet B light therapy and antidepressants were prescribed more widely as third-line of therapy (Supplementary Figure S6).

## Discussion

This real-world study aimed to establish current diagnosis, management, and unmet clinical needs of patients on hemodialysis with CKD-aP. The survey data showed that nephrologist-perceived prevalence of CKD-aP was considerably lower (43.6%) than the percentage of patients reporting itching in DOPPS (2012–2015; 68%), potentially reflecting underreporting of itching by patients to health care professionals.^2^

However, the estimated incidence of mild, moderate, and severe CKD-aP by nephrologists and PRFs was similar, and reflected those in DOPPS (2012–2015); for example, the percentage of CKD-aP patients with mild itching was estimated by nephrologists to be 40% and reported in 32% of PRFs, whereas at least moderately bothersome itching was reported by 55% of CKD-aP patients in DOPPS.^2^ Similarities between nephrologist-estimated incidence of CKD-aP severity and pruritus severity stated in the PRFs in the current study may indicate an increased awareness of CKD-aP by nephrologists, particularly when compared to previous DOPPS data reporting failure of medical directors to accurately estimate the prevalence of CKD-aP in 69% of facilities.^2^ However, caution should be taken when making such comparisons, because PRFs were selected by nephrologists following completion of the questionnaire, potentially resulting in selection bias or overrepresentation of patients with severe pruritus who are more likely to be treated by a nephrologist.

Although most nephrologists used a mild-moderate-severe classification for itch severity, this report highlights a lack of standardization, with low uptake of clinical itch scales. As a result, a large proportion of nephrologists noted a need for new guidelines and a consistent international scale for CKD-aP.

Data from both the survey and PRFs highlighted frequent use of moisturizers or emollients, antihistamines, and gabapentinoids. Oral antihistamines were frequently employed as a first-line pharmacologic agent, whereas gabapentinoids were generally reserved for second-line or third-line treatment, possibly reflecting that although gabapentinoids are generally considered safe and effective, there is an increased risk of mental changes, falls, and fractures with these treatments.^5^ Prescription of gabapentinoids, estimated by nephrologists and in PRFs, was higher than previously reported by DOPPS (2012–2015), in which over half of medical directors never prescribed gabapentin for pruritus.^2^ This increased uptake of gabapentinoids may reflect the publication of reviews and guidelines supporting their effective use.^5^, ^6^, ^7^ Opioid receptor modulators, such as nalfurafine hydrochloride, were used least frequently, reflecting their variable efficacy in clinical trials, associated central nervous system side effects, and lack of European approval.^5^, ^6^, ^7^ The variation observed in first-line, second-line, and third-line treatment patterns aligns with numerous reports demonstrating a lack of robust data-supported guidelines for CKD-aP.^5^, ^6^, ^7^, ^8^

The survey revealed a low level of physician satisfaction with current therapies, with less than half of nephrologists highly or moderately satisfied with the tolerability of treatments listed, and less than one-third highly or moderately satisfied with their impact on patient QoL. Low satisfaction with current therapies may reflect the frequent use of antihistamines, which have been shown to be ineffective in randomized clinical trials.^1^ It is worth noting, however, that because this study revealed low usage of standardized methods to quantify itch, comments by respondents regarding treatment effectiveness, tolerability, and impact on QoL are unlikely to be based on quantitative data.

These findings are limited by the relatively small number of nephrologists surveyed, PRFs collected, and countries included as well as the restriction of respondents to nephrologists only. However, this study reinforces the emphasis on pruritus as a symptom of core importance to patients with CKD.

## Disclosure

JB reports receiving honoraria for speaker engagements and/or travel from Astellas, AstraZeneca, Diaverum, and Vifor Pharma. SW and SD report being employees of CSL Vifor. BS reports receiving honoraria for speaker engagements and/or travel from Astellas, AstraZeneca, Vifor Pharma, Amgen, Novartis, Bayer, Boehringer Ingelheim, and Alexion.

## Supplementary Material

**Supplementary Methods.**

**Figure S1.** Nephrologists’perception of prevalence, classification, and severity of CKD-aP: (A) nephrologist-perceived prevalence of CKD-aP in patients treated in the past month, by country and overall, (B) percentage of nephrologists using a mildmoderate-severe classification system for CKD-aP, by country and overall, and (C) percentage of patients with mild, moderate, and severe CKD-aP as estimated by nephrologists.

**Figure S2.** Nephrologists’ use of itch scales: (A) percentage of nephrologists using any itch scales to determine the severity of a patient’s CKD-aP and (B) percentage of nephrologists using specific itch scales to determine the severity of a patient’s CKD-aP.

**Figure S3.** Nephrologist-perceived frequency of CKD-aP treatment use in each of the countries studied: (A) France, (B) Germany, (C) Italy, (D) Spain, (E) UK, and (F) Australia.

**Figure S4.** Nephrologists’ perception of needs for CKD-aP therapy: (A) nephrologists’ level of agreement with the statements “CKD-aP represents a minor concern when considering the broader context of a patient’s CKD” (i) and “There is a need for new treatments specifically designed to address CKD-aP” (ii) and (B) nephrologist-perceived level of improvement needed when thinking about future treatments for CKD-aP.

**Figure S5.** Severity, location, and impacts of CKD-aP according to PRF data: (A) percentage of patients with mild, moderate, and severe CKD-aP, (B) length of time patients suffered with CKD-aP, and (C) percentage of patients reporting itch affecting different parts of the body.

**Figure S6.** Percentage of mild (A), moderate (B), and severe (C) patients receiving each CKD-aP treatment for first, second, and third lines of therapy, according to PRF data.

**Table S1.** Regional distribution of nephrologists surveyed.

**Table S2.** Factors associated with mild, moderate, and severe CKD-aP classification by nephrologists surveyed.

**Table S3.** Nephrologists’ satisfaction with CKD-aP treatments.
