# Supplementary material for: Current Practices in CKD-Associated Pruritus: International Nephrologist Survey
Source: Kidney Int Rep. 2023 Apr 7;8(7):1455–9. doi: 10.1016/j.ekir.2023.04.003 (PMC10334356; doi:10.1016/j.ekir.2023.04.003)
Supplement: Supplemental File (PDF) [file mmc1.pdf]

**SUPPLEMENTAL DATA**

**Title:** Current Practices in Chronic Kidney Disease-Associated Pruritus: International Nephrologist Survey

**Authors:** James O. Burton, MD[1], Sebastian Walpen, PhD[2], Sandrine Danel, MSc[2], Bernd Schröppel, MD[3]

# Contents

METHODS ........................................................................................ 4

SUPPLEMENTAL TABLES AND FIGURES ........................................................ 6

Supplemental Table S1. Regional distribution of nephrologists surveyed .................. 6

Supplemental Table S2. Factors associated with mild, moderate, and severe CKD-aP classification by nephrologists surveyed .................................... 8

Supplemental Table S3. Nephrologists' satisfaction with CKD-aP treatments ........... 9

Supplemental Figure S1. Nephrologists' perception of prevalence, classification, and severity of CKD-aP: (a) nephrologist-perceived prevalence of CKD-aP in patients treated in the past month, by country and overall, (b) percentage of nephrologists using a mild-moderate-severe classification system for CKD-aP, by country and overall, and (c) percentage of patients with mild, moderate, and severe CKD-aP as estimated by nephrologists ................................................................. 10

Supplemental Figure S2. Nephrologists' use of itch scales: (a) percentage of nephrologists using any itch scales to determine the severity of a patient's CKD-aP and (b) percentage of nephrologists using specific itch scales to determine the severity of a patient's CKD-aP ................... 12

Supplemental Figure S3. Nephrologist-perceived frequency of CKD-aP treatment use in each of the countries studied: (a) France, (b) Germany, (c) Italy, (d) Spain, (e) UK, and (f) Australia........................... 14

Supplemental Figure S4. Nephrologists' perception of needs for CKD-aP therapy: (a) nephrologists' level of agreement with the statements "CKD-aP represents a minor concern when considering the broader context of a patient's CKD" (i) and "There is a need for new treatments specifically designed to address CKD-aP" (ii) and (b) nephrologist-perceived level of improvement needed when thinking about future treatments for CKD-aP ...................................................... 18

Supplemental Figure S5. Severity, location, and impacts of CKD-aP according to PRF data: (a) percentage of patients with mild, moderate, and severe CKD-aP, (b) length of time patients suffered with CKD-aP, and (c) percentage of patients reporting itch affecting different parts of the body ........................................................................................ 20

Supplemental Figure S6. Percentage of mild (a), moderate (b), and severe (c) patients receiving each CKD-aP treatment for first, second and third lines of therapy, according to PRF data ................................................ 22

SUPPLEMENTAL APPENDIX ........................................................................ 24

## METHODS

### ***Physician perception survey conduct and analysis***

Quantitative data were collected between May 2020 and July 2020 via a 20-minute online physician survey and collection of patient record forms (PRFs), shown in the **Supplemental Appendix**. Respondents were randomly and anonymously selected among the Market Research agency's panel. Responses from nephrologists were included if the following inclusion criteria were met; responsible for the treatment of ≥50 patients with chronic kidney disease (CKD) on hemodialysis (HD) per month (>30 per month in Australia), including ≥4 patients with CKD-associated pruritus (CKD-aP); 3–30 years' clinical practice experience; decision maker for the related condition treatment and spend majority of time in direct patient care; reported no conflicts of interest. The survey was conducted in the local language, the identity of the sponsor was blinded, and respondents were anonymized. Respondents received compensation directly from the Market Research agency that was proportional to the time spent filling in the survey and independent from answers provided. Respondents' level of agreement was assessed using a 7-point scale, from 1 (do not agree at all) to 7 (strongly agree). Respondent's satisfaction was assessed using a 7-point scale, from 1 (extremely dissatisfied) to 7 (extremely satisfied) and respondent's perceived frequency of treatment use was assessed using a 4-point scale including "do not use", "use rarely", "use sometimes" and "use often".

### ***Patient record form data analysis***

PRF data were selected from patients with CKD-aP who had physician consultations in the previous 3 months.

### *Data analysis*

Nephrologists were asked to provide the number of patients with CKD on center-based HD that they had personally treated in the past month, and how many of these patients experienced CKD-aP. Averages were calculated and presented as percentages.

Criteria used to determine patient severity are described in **Supplemental Table S2**. Selection of 7 or 1 on the agreement scale was presented as “strongly agreed” or “strongly disagreed”, respectively. Selection of 6 was presented as “moderately agreed”. Scores 7 and 6 on the satisfaction scale were presented as “highly satisfied” and “moderately satisfied”, respectively. Percentages were rounded to the nearest whole percent.

## SUPPLEMENTAL TABLES AND FIGURES

Supplemental Table S1. Regional distribution of nephrologists surveyed

| France (n=50) | Germany (n=56) | Italy (n=58) | Spain (n=55) | UK (n=52) | Australia (n=30) |
|---|---|---|---|---|---|
| Île-de-France **(26%)** | Nordrhein-Westfalen **(34%)** | Latium **(29%)** | Madrid **(38%)** | Greater London **(25%)** | New South Wales **(50%)** |
| Hauts-de-France **(12%)** | Hessen **(14%)** | Lombardia **(12%)** | Andalucía **(15%)** | East Midlands **(14%)** | Victoria **(33%)** |
| Provence-Alpes-Côte d'Azur **(12%)** | Freie und Hansestadt Hamburg **(11%)** | Sicilia **(12%)** | Comunidad Valenciana **(13%)** | Northwest England **(14%)** | Queensland **(7%)** |
| Nouvelle-Aquitaine **(10%)** | Baden-Württemberg **(9%)** | Puglia **(10%)** | Asturias **(7%)** | Southeast England **(14%)** | Western Australia **(7%)** |
| Auvergne Rhône-Alpes **(10%)** | Freistaat Bayern **(7%)** | Campania **(10%)** | Castilla la Mancha **(7%)** | West Midlands **(10%)** | Northern Territory **(3%)** |
| Bourgogne Franche-Comté **(8%)** | Niedersachsen **(5%)** | Emilia-Romagna **(9%)** | Cataluña **(6%)** | Scotland **(10%)** | |
| Pays de la Loire **(6%)** | Berlin **(5%)** | Abruzzo **(4%)** | Aragón **(4%)** | Yorkshire and the Humber **(8%)** | |
| Grand Est **(6%)** | Mecklenburg-Vorpommern **(4%)** | Piemonte **(4%)** | Castilla y León **(4%)** | Southwest England **(4%)** | |
| Centre Val de Loire **(4%)** | Sachsen-Anhalt **(4%)** | Marche / Calabria / Liguria / Friuli-Venezia Giulia / Toscana / Veneto **(all 2%)** | Extremadura / Baleares / Navarra / País Vasco **(all 2%)** | Northeast England **(4%)** | |
| Occitanie **(4%)** | Freistaat Sachsen / Rheinland-Pfalz / Saarland / Brandenburg **(all 2%)** | | | | |

| | | | | | |
|---|---|---|---|---|---|
| Bretagne **(2%)** | | | | | |

N=301; data from survey, S9.

Supplemental Table S2. Factors associated with mild, moderate, and severe CKD-aP classification by nephrologists surveyed

| Classification | Factors used by nephrologists (% of nephrologists) |
|---|---|
| Mild | • Occasional itching (86%) |
| Moderate | • Continuous itching (54%)<br>• Itch persists despite:<br>○ topical treatments (70%)<br>○ dialysis optimization (58%)<br>○ antihistamines (60%) |
| Severe | • Itch persists despite gabapentinoids (77%)<br>• Visible scratch marks (66%)<br>• Skin abrasions (78%)<br>• Impact on:<br>○ sleep (80%)<br>○ daily activities (77%)<br>○ mood (77%) |

N=301; data from survey, Q2B. Data shown represent the percentages of nephrologists associating each characteristic with that severity level.

CKD-aP, chronic kidney disease-associated pruritus.

Supplemental Table S3. Nephrologists' satisfaction with CKD-aP treatments

| | | Rating | | | | | | |
|---|---|---|---|---|---|---|---|---|
| | | 1 | 2 | 3 | 4 | 5 | 6 | 7 |
| Topical / Oral antihistamines (n=299) | Efficacy in monotherapy | 4% | 11% | 17% | 25% | 22% | 19% | 3% |
| | Tolerability | 0% | 3% | 8% | 17% | 28% | 33% | 11% |
| | Impact on patient's QoL | 1% | 5% | 16% | 24% | 29% | 17% | 8% |
| Topical / Oral corticosteroids (n=278) | Efficacy in monotherapy | 1% | 12% | 13% | 25% | 27% | 17% | 4% |
| | Tolerability | 2% | 8% | 14% | 27% | 27% | 18% | 4% |
| | Impact on patient's QoL | 1% | 7% | 15% | 27% | 23% | 22% | 5% |
| Gabapentinoids (n=289) | Efficacy in monotherapy | 1% | 4% | 18% | 23% | 32% | 19% | 4% |
| | Tolerability | 0% | 8% | 20% | 28% | 24% | 14% | 6% |
| | Impact on patient's QoL | 0% | 4% | 14% | 26% | 33% | 18% | 6% |
| | Convenient mode of administration[a] | 0% | 2% | 8% | 22% | 33% | 27% | 8% |
| Antidepressants / Anxiolytics / Sedatives (n=282) | Efficacy in monotherapy | 1% | 13% | 23% | 27% | 22% | 13% | 2% |
| | Tolerability | 0% | 10% | 21% | 25% | 27% | 15% | 3% |
| | Impact on patient's QoL | 0% | 6% | 20% | 28% | 26% | 17% | 4% |
| | Convenient mode of administration[a] | 0% | 1% | 11% | 24% | 32% | 27% | 5% |
| Opioid receptor modulators (n=261) | Efficacy in monotherapy | 0% | 7% | 13% | 29% | 29% | 19% | 2% |
| | Tolerability | 2% | 9% | 19% | 32% | 23% | 12% | 3% |
| | Impact on patient's QoL | 1% | 6% | 16% | 25% | 32% | 18% | 3% |
| | Convenient mode of administration[a] | 0% | 4% | 12% | 26% | 35% | 20% | 3% |
| UVB therapy (n=266) | Efficacy in monotherapy | 2% | 7% | 14% | 29% | 30% | 14% | 5% |
| | Tolerability | 0% | 5% | 11% | 25% | 31% | 23% | 5% |
| | Impact on patient's QoL | 0% | 5% | 13% | 21% | 37% | 18% | 6% |
| | Convenient mode of administration[a] | 8% | 18% | 19% | 16% | 22% | 15% | 3% |

N=261–299 (based on those answering survey with experience of each class of CKD-aP treatments); data from survey, Q9. [a]Not asked for: topical/oral antihistamine or corticosteroids.

CKD-aP, chronic kidney disease-associated pruritus; QoL, quality of life; UVB, ultraviolet B.

Supplemental Figure S1. Nephrologists' perception of prevalence, classification, and severity of CKD-aP: (a) nephrologist-perceived prevalence of CKD-aP in patients treated in the past month, by country and overall, (b) percentage of nephrologists using a mild-moderate-severe classification system for CKD-aP, by country and overall, and (c) percentage of patients with mild, moderate, and severe CKD-aP as estimated by nephrologists

a

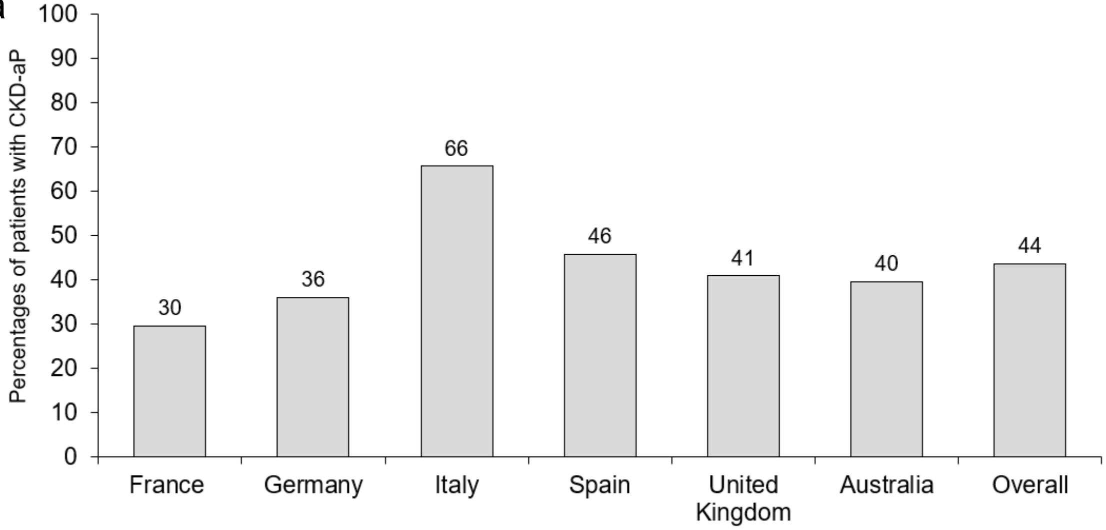


b

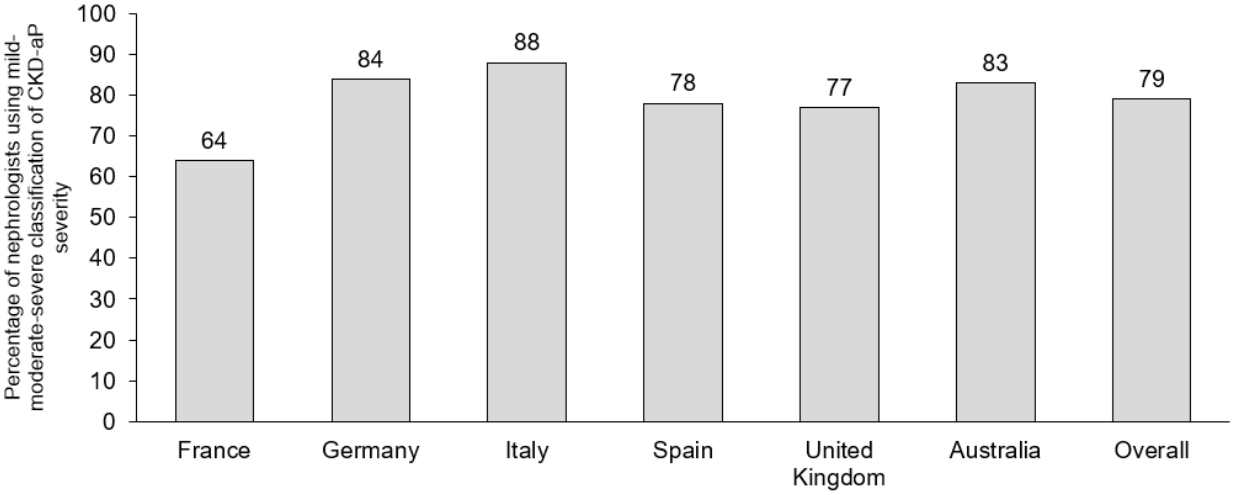

c

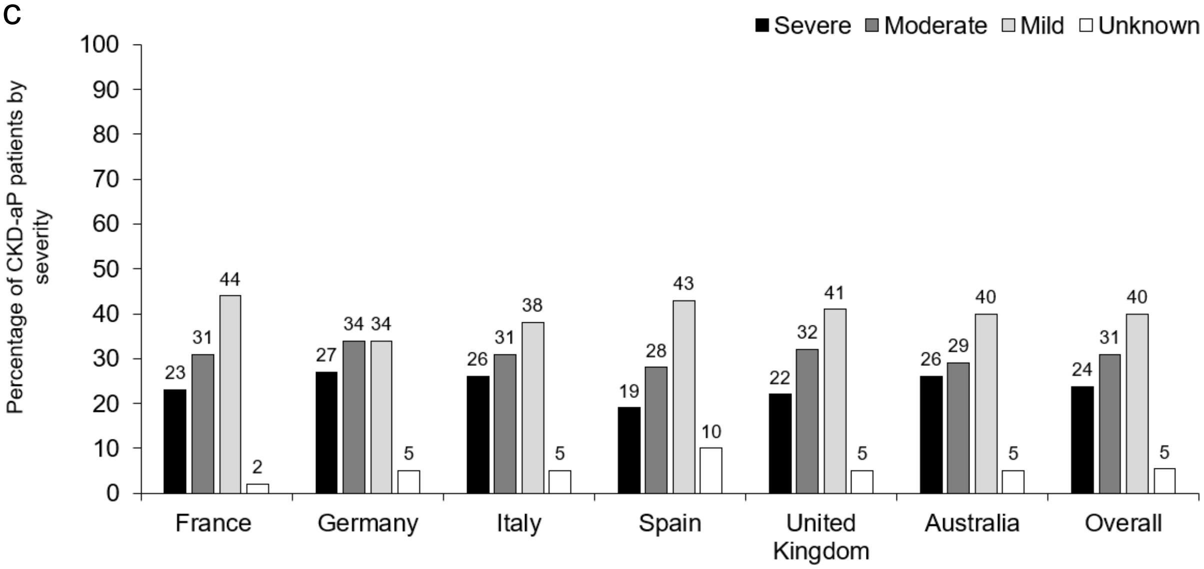


(a) N=301; data from survey, S5.

(b) N=301; data from survey, Q2A.

(c) N=301; data from survey, Q2C.

CKD-aP, chronic kidney disease-associated pruritus.

Supplemental Figure S2. Nephrologists' use of itch scales: (a) percentage of nephrologists using any itch scales to determine the severity of a patient's CKD-aP and (b) percentage of nephrologists using specific itch scales to determine the severity of a patient's CKD-aP

a

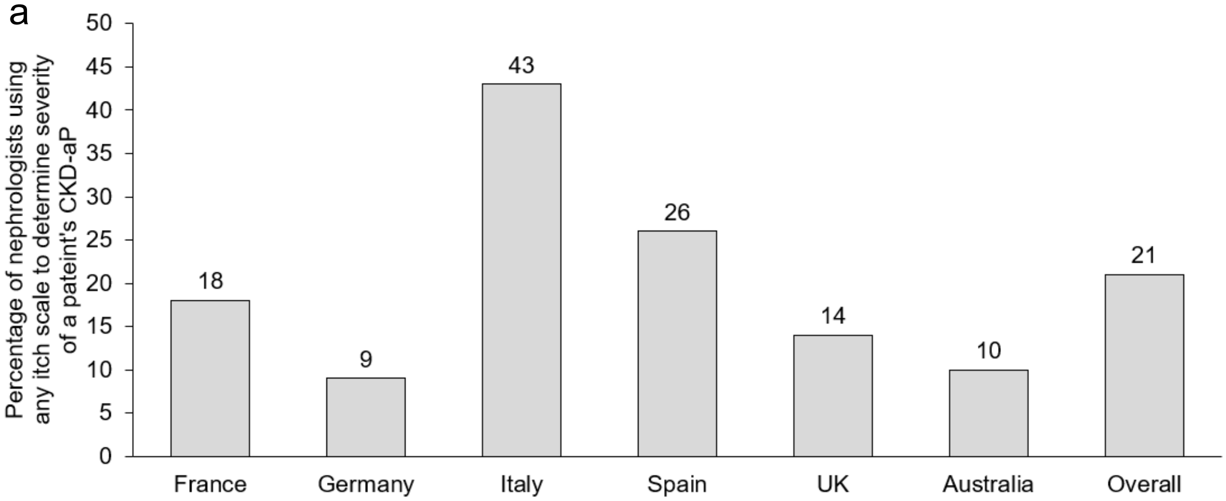

b

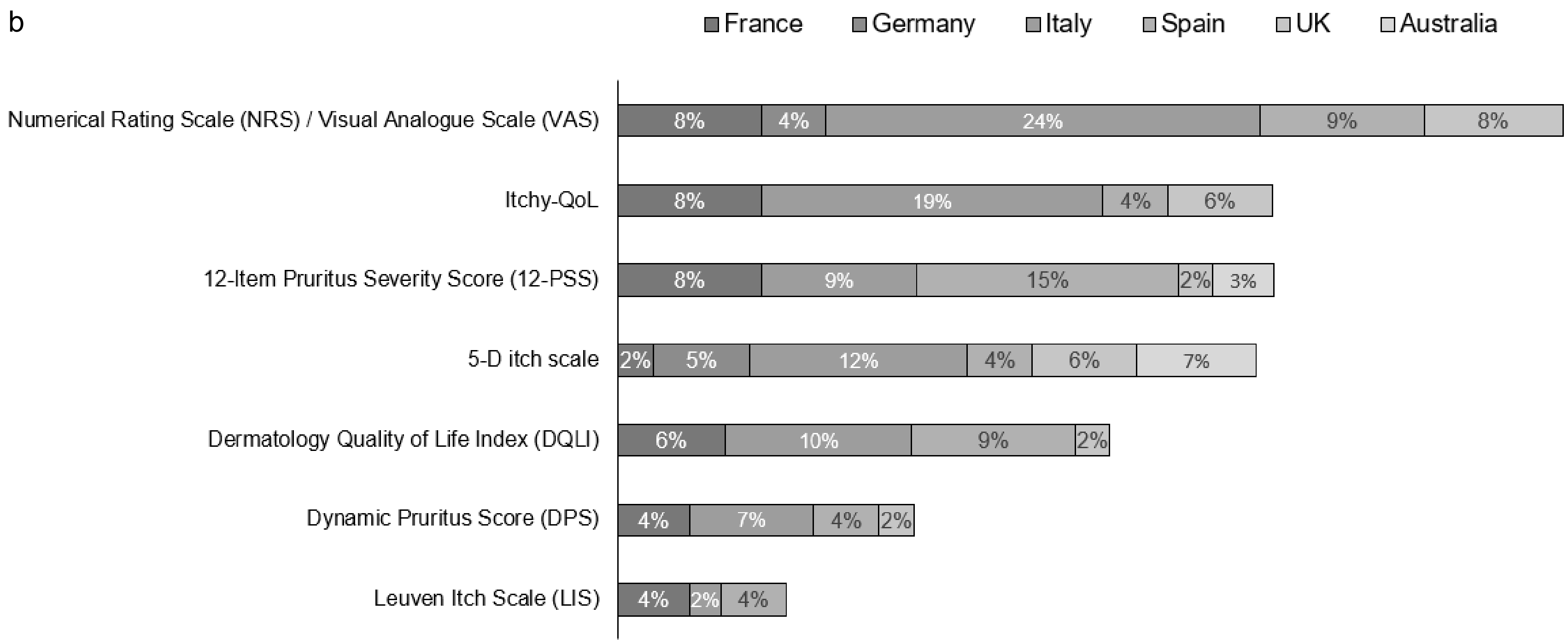


(a) N=301; data from survey, Q2F.

(b) N=63 (France, n=9; Germany, n=5; Italy, n=25; Spain, n=14; UK, n=7; Australia, n=3); data from survey, Q2G.

CKD-aP, chronic kidney disease-associated pruritus; QoL, quality of life.

Supplemental Figure S3. Nephrologist-perceived frequency of CKD-aP treatment use in each of the countries studied: (a) France, (b) Germany, (c) Italy, (d) Spain, (e) UK, and (f) Australia

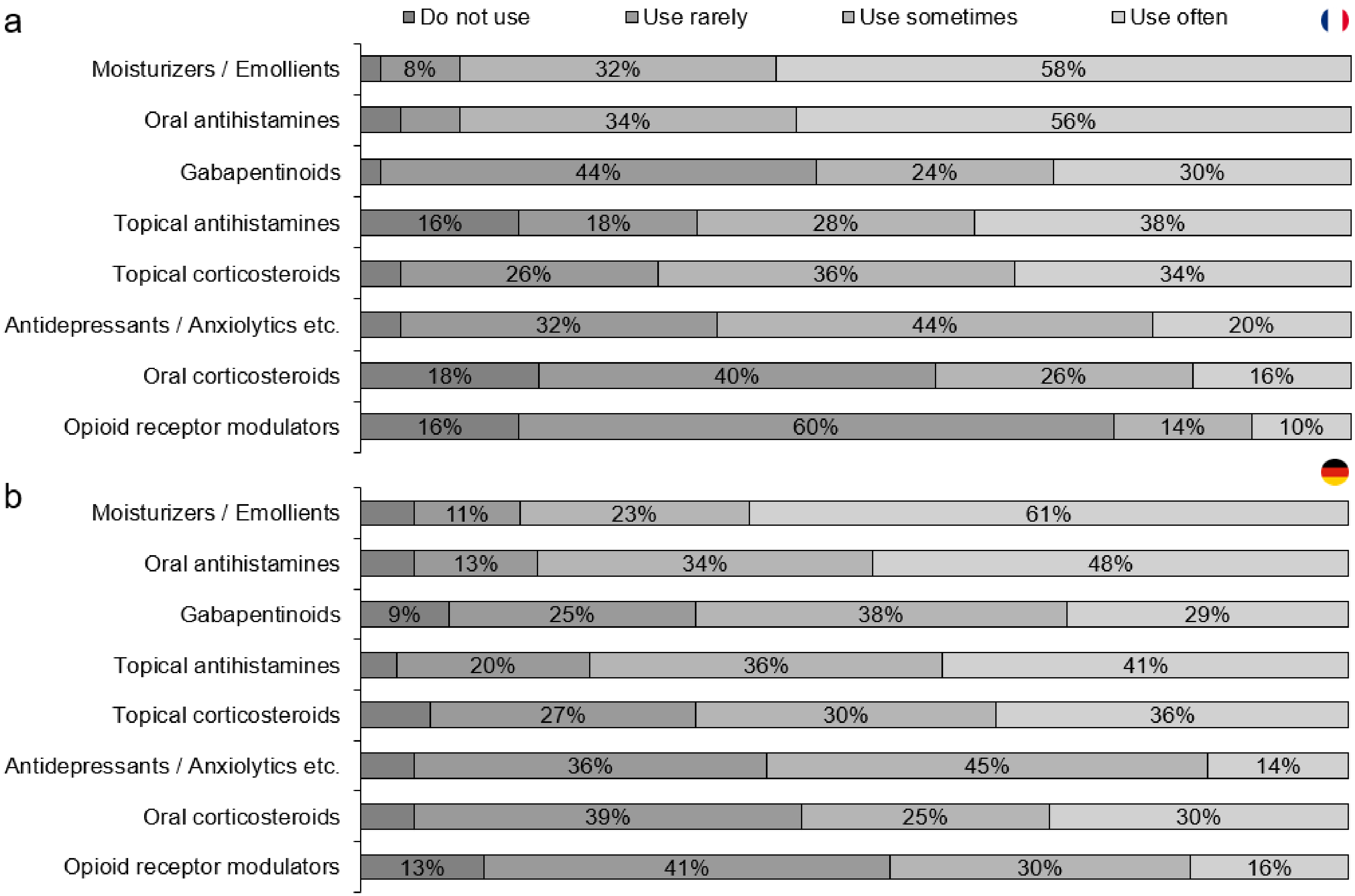

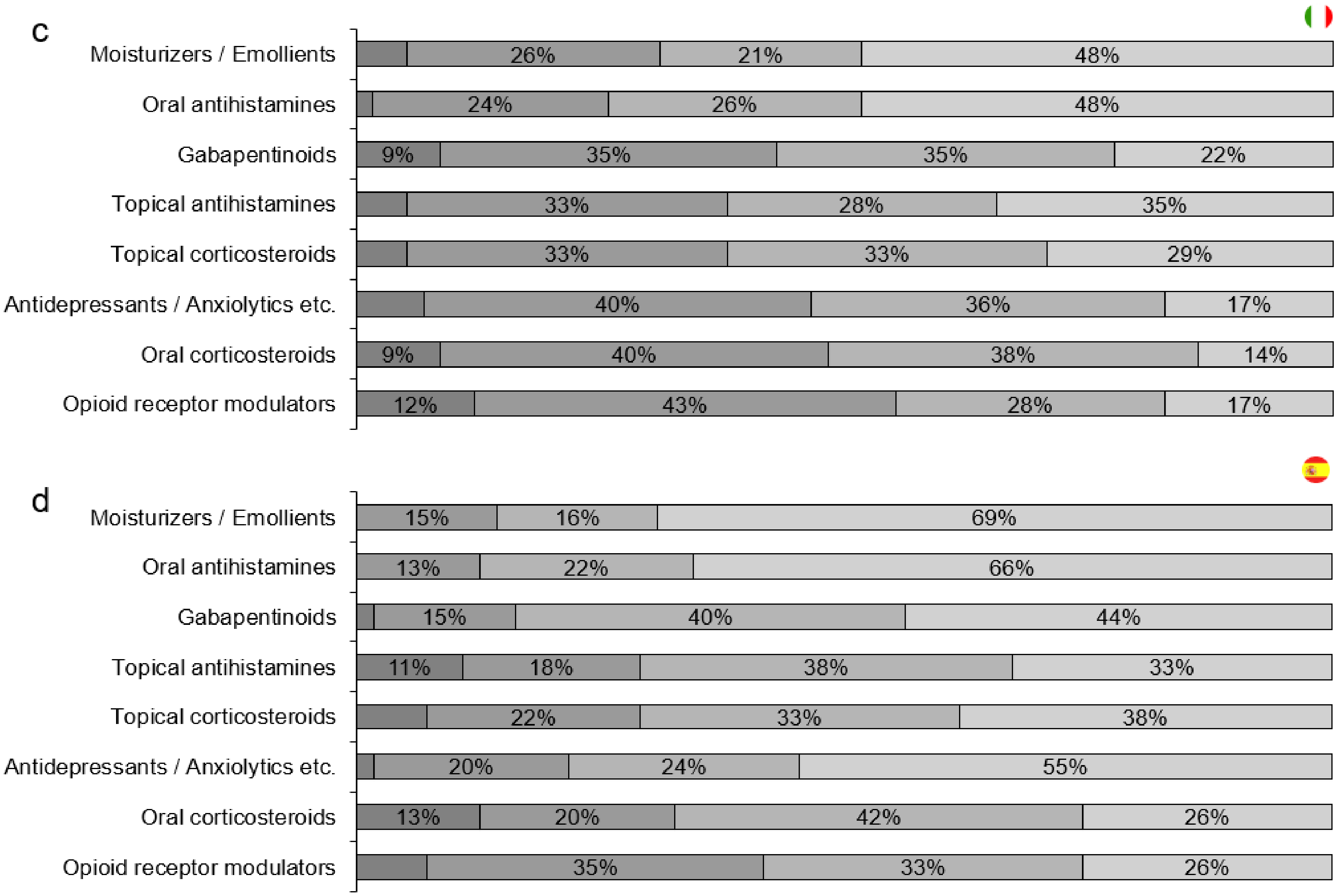
c
Moisturizers / Emollients
26%
21%
48%
Oral antihistamines
24%
26%
48%
Gabapentinoids
9%
35%
35%
22%
Topical antihistamines
33%
28%
35%
Topical corticosteroids
33%
33%
29%
Antidepressants / Anxiolytics etc.
40%
36%
17%
Oral corticosteroids
9%
40%
38%
14%
Opioid receptor modulators
12%
43%
28%
17%
d
Moisturizers / Emollients
15%
16%
69%
Oral antihistamines
13%
22%
66%
Gabapentinoids
15%
40%
44%
Topical antihistamines
11%
18%
38%
33%
Topical corticosteroids
22%
33%
38%
Antidepressants / Anxiolytics etc.
20%
24%
55%
Oral corticosteroids
13%
20%
42%
26%
Opioid receptor modulators
35%
33%
26%

e

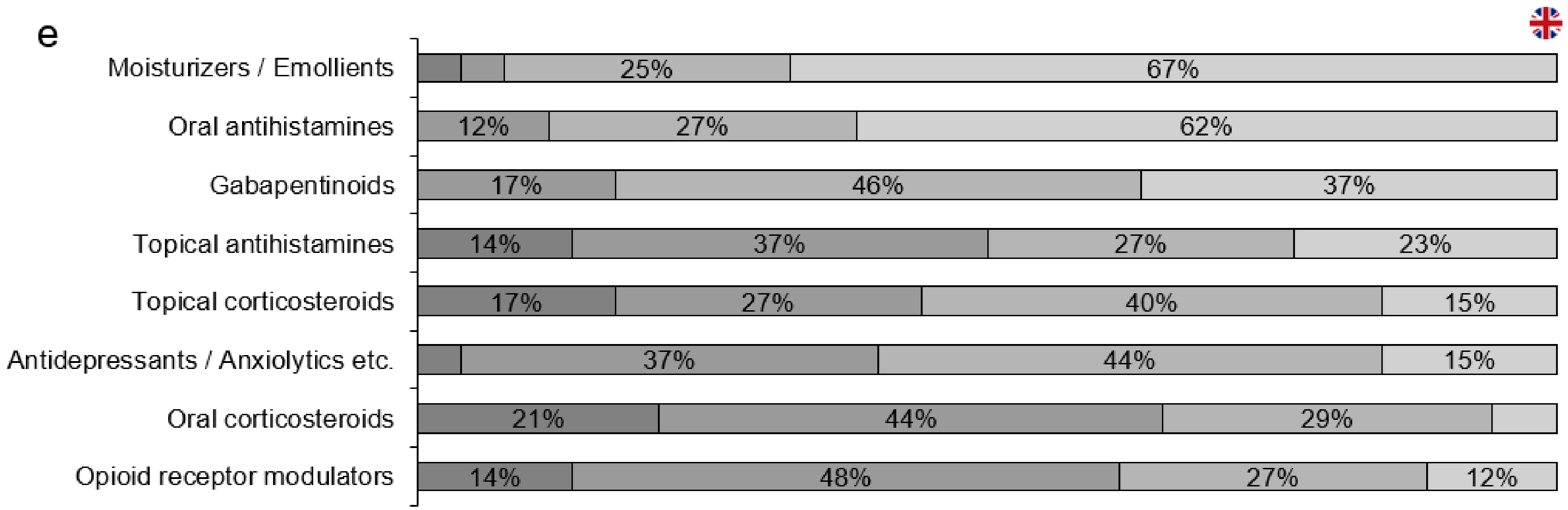
Moisturizers / Emollients
25%
67%
Oral antihistamines
12%
27%
62%
Gabapentinoids
17%
46%
37%
Topical antihistamines
14%
37%
27%
23%
Topical corticosteroids
17%
27%
40%
15%
Antidepressants / Anxiolytics etc.
37%
44%
15%
Oral corticosteroids
21%
44%
29%
Opioid receptor modulators
14%
48%
27%
12%

f

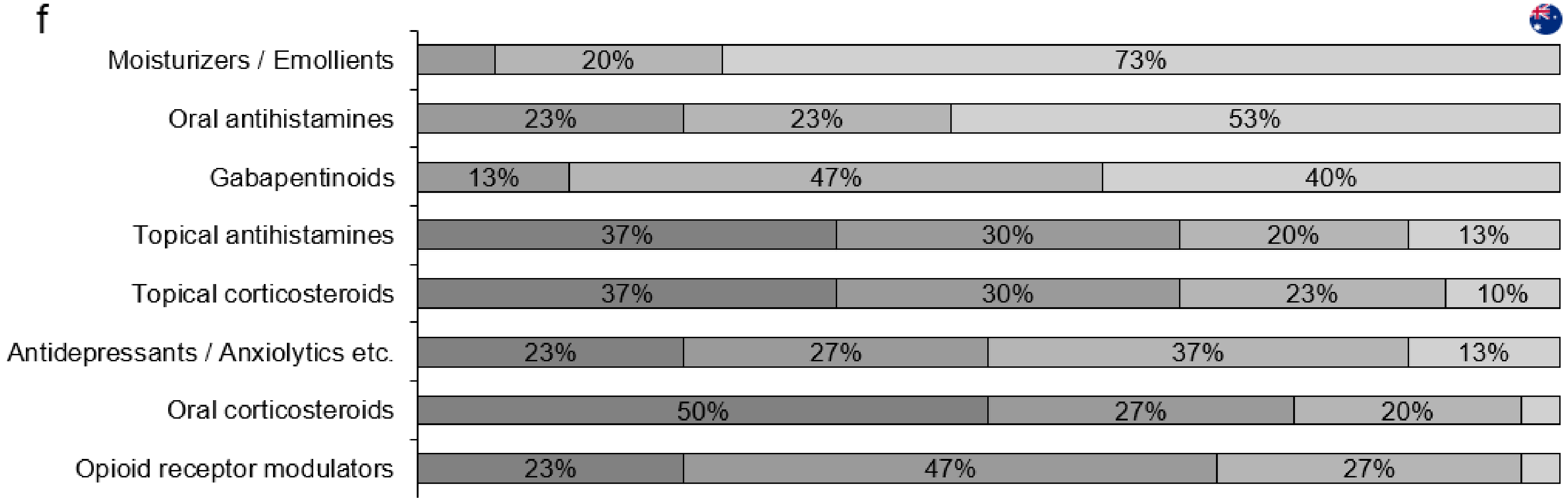
Moisturizers / Emollients
20%
73%
Oral antihistamines
23%
23%
53%
Gabapentinoids
13%
47%
40%
Topical antihistamines
37%
30%
20%
13%
Topical corticosteroids
37%
30%
23%
10%
Antidepressants / Anxiolytics etc.
23%
27%
37%
13%
Oral corticosteroids
50%
27%
20%
Opioid receptor modulators
23%
47%
27%

France, n=50; Germany, n=56; Italy, n=58; Spain, n=55; UK, n=52; Australia, n=30; data from survey, Q3. Data labels <8% are hidden from chart for clarity.

CKD-aP, chronic kidney disease-associated pruritus.

Supplemental Figure S4. Nephrologists' perception of needs for CKD-aP therapy: (a) nephrologists' level of agreement with the statements "CKD-aP represents a minor concern when considering the broader context of a patient's CKD" (i) and "There is a need for new treatments specifically designed to address CKD-aP" (ii) and (b) nephrologist-perceived level of improvement needed when thinking about future treatments for CKD-aP

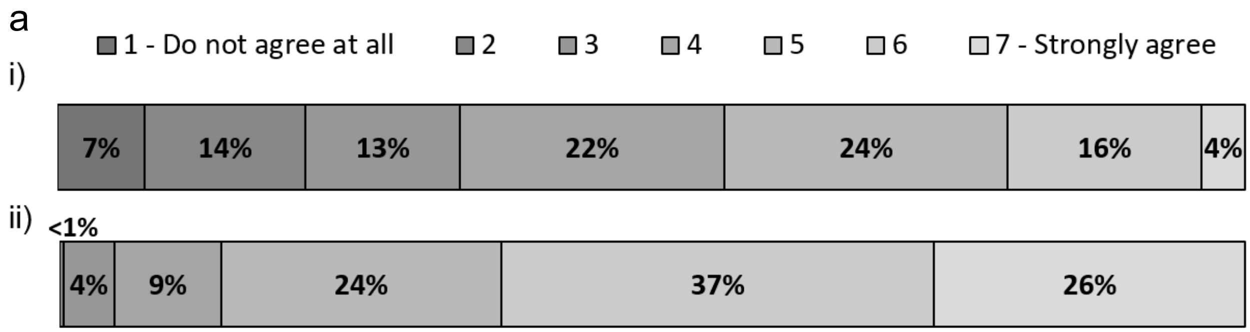

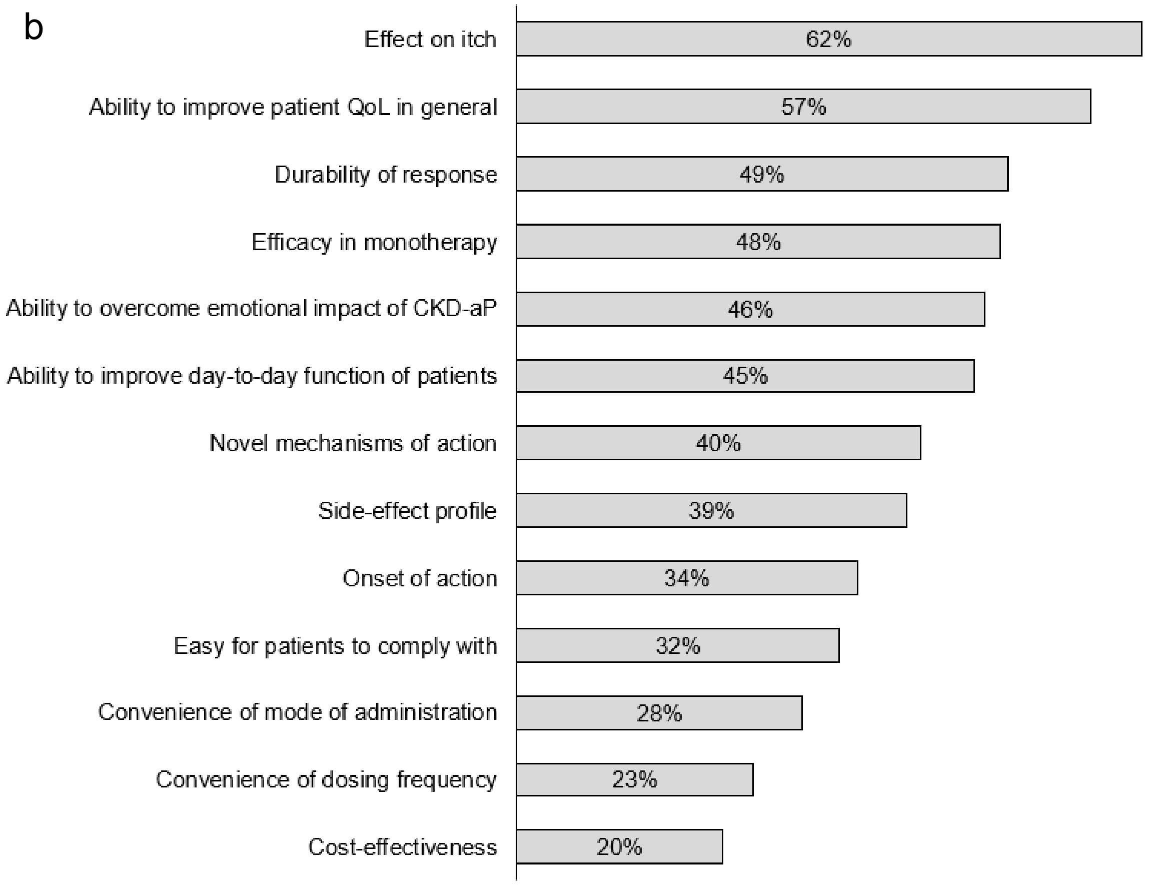


(a) N=301; data from survey, Q10. (aii) Less than 1% of respondents selected 1 or 2 on the agreement scale.

(b) N=301; data from survey, Q11.

CKD-aP, chronic kidney disease-associated pruritus; QoL, quality of life.

Supplemental Figure S5. Severity, location, and impacts of CKD-aP according to PRF data: (a) percentage of patients with mild, moderate, and severe CKD-aP, (b) length of time patients suffered with CKD-aP, and (c) percentage of patients reporting itch affecting different parts of the body

a

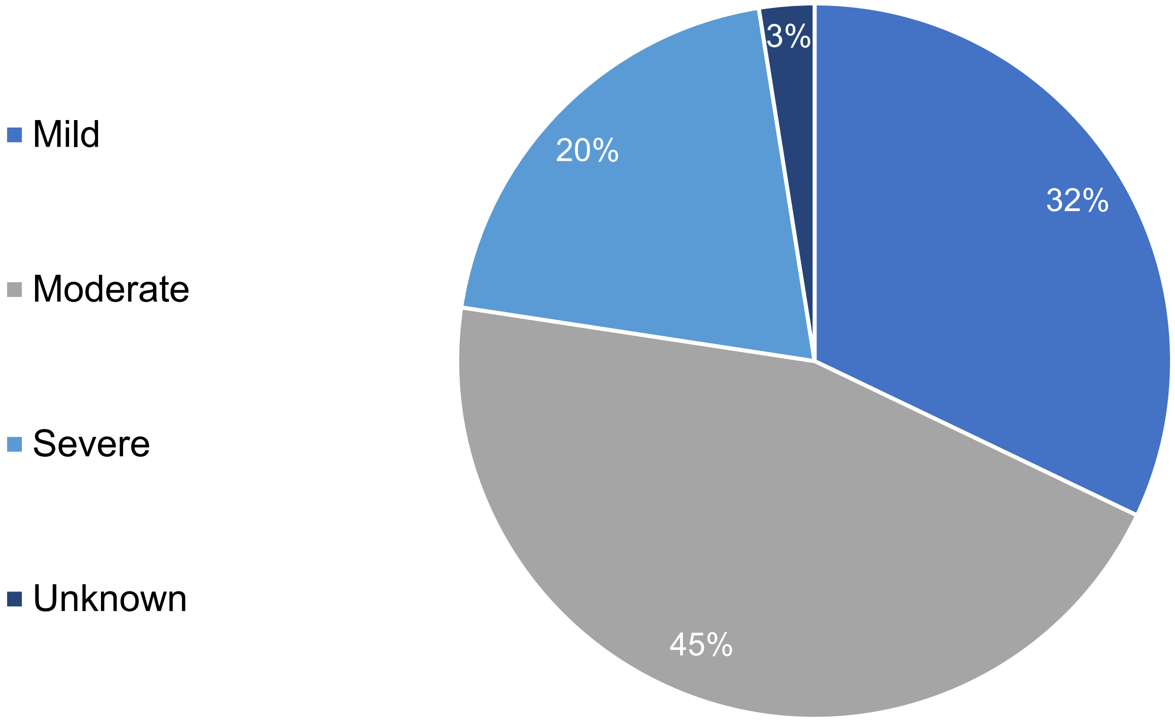

b

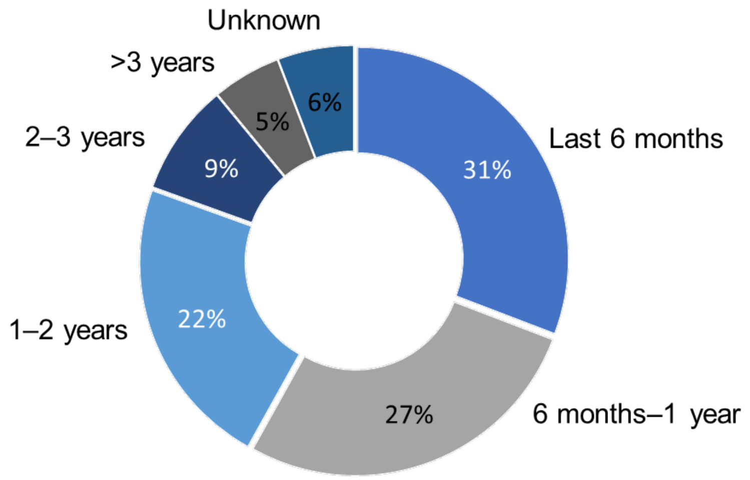


c

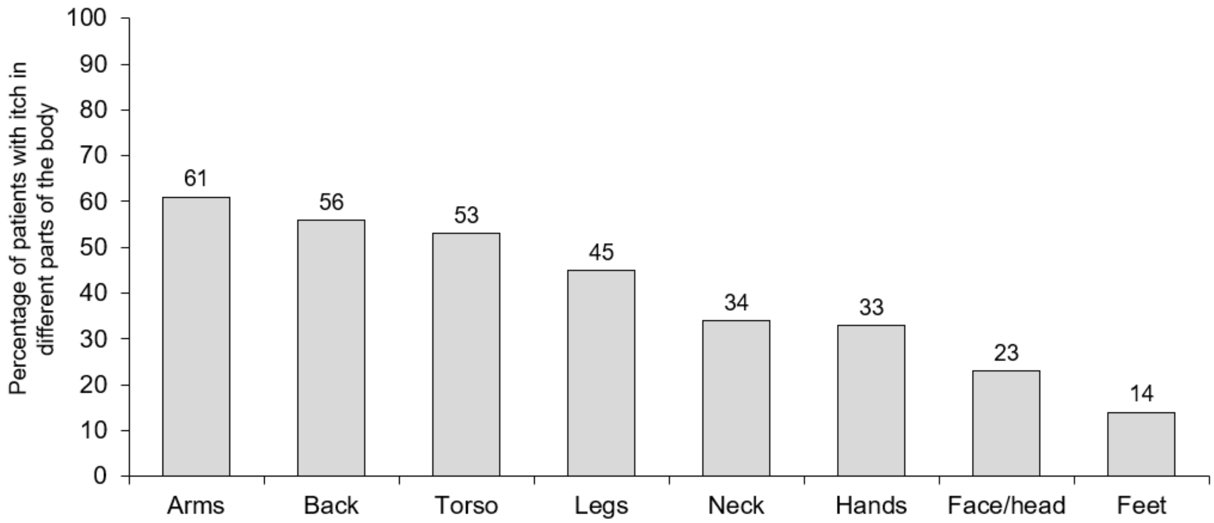


N=1,435, data from PRFs.

CKD-aP, chronic kidney disease-associated pruritus; PRF patient record form.

Supplemental Figure S6. Percentage of mild (a), moderate (b), and severe (c) patients receiving each CKD-aP treatment for first, second and third lines of therapy, according to PRF data

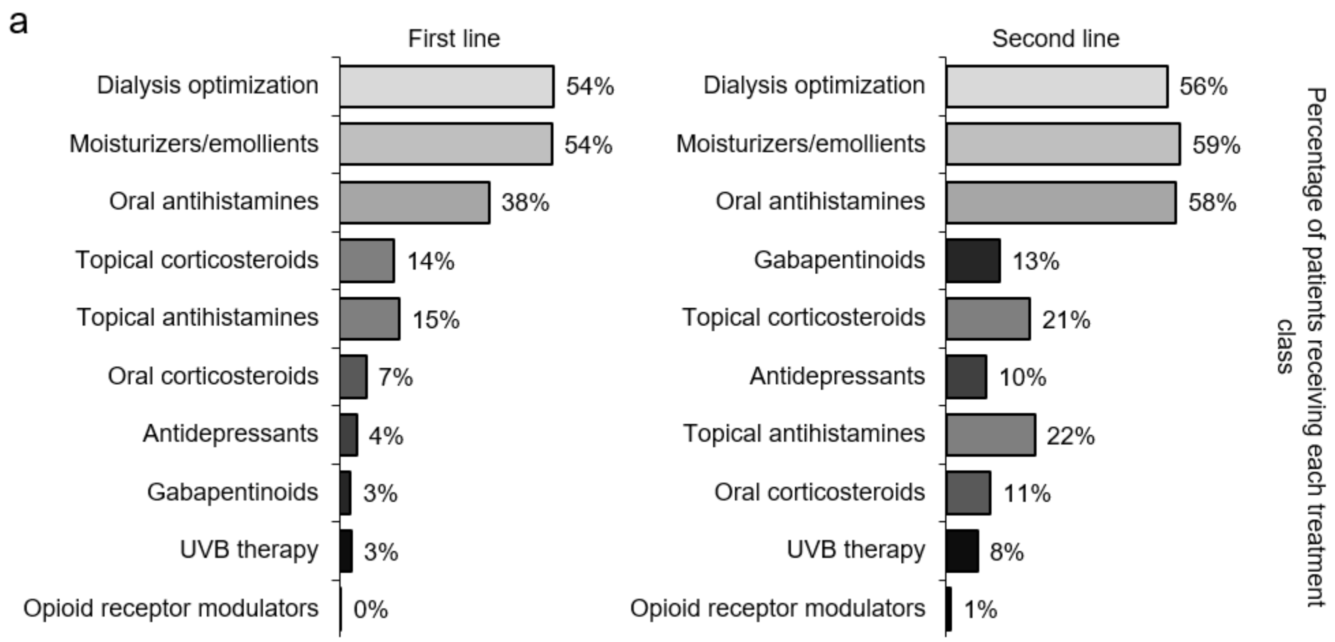

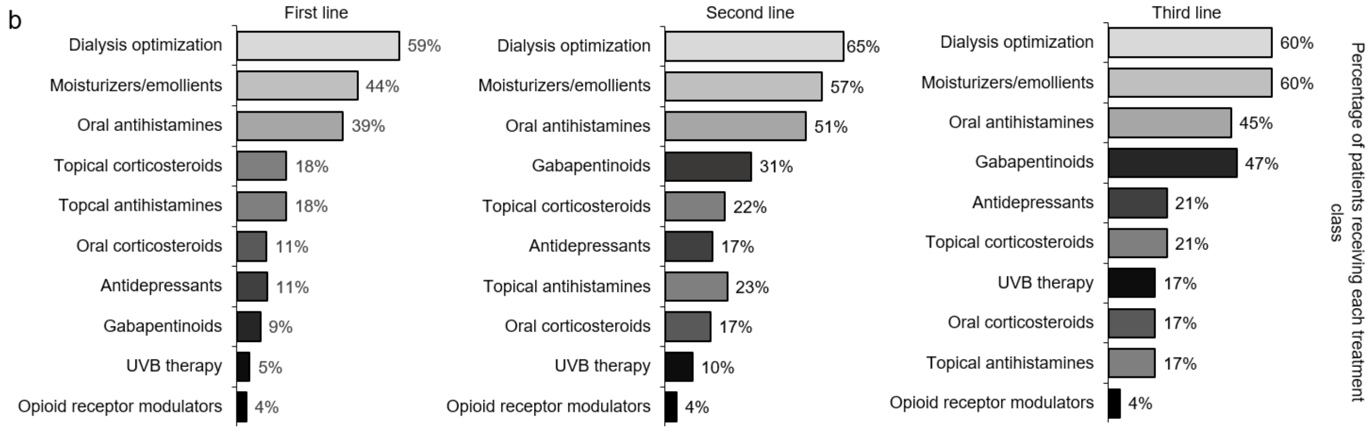


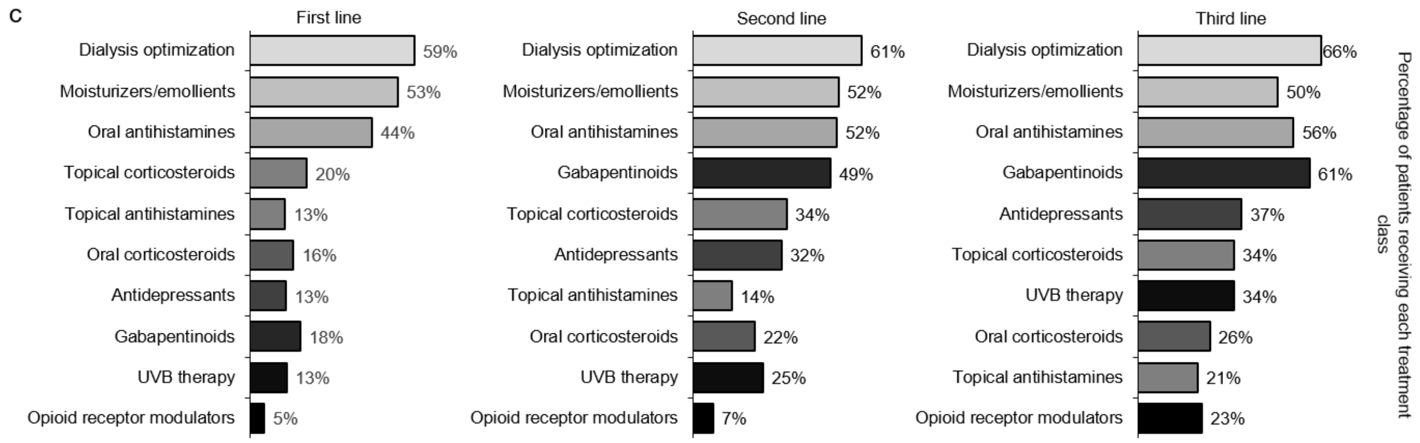


(a) First line, n=460; second line, n=90; data from PRFs.

(b) First line, n=650; second line, n=261; third line, n=47; data from PRFs.

(c) First line, n=289; second line, n=167; third line, n=70; data from PRFs.

Percentage of patients receiving each treatment shown includes treatments given as either a monotherapy or combination therapy.

CKD-aP, chronic kidney disease-associated pruritus; PRF, patient report form; UVB, ultraviolet B.

## SUPPLEMENTAL APPENDIX

### MARKET RESEARCH: "CKD-aP in HD-CKD Patients"

### Online interviews with Nephrologists in EU5

| NON-PROMOTIONAL MATERIAL | |
|---|---|
| Objectives | Quantification of local / actual management of CKD-aP among HD-CKD patients<br>• Treatment choices based on patient profiles (e.g. severity)<br>• Attitudes towards management of CKD-aP, including unmet needs |
| Fieldwork | Q1 2020 |
| Sample | Nephrologists, decision makers for CKD-aP treatment in HD-CKD:<br>n=50 per market (must achieve n=300 in total and at least n=30 in AU) |
| Methodology | 20-minute online survey<br>40-minute PRF task (min. 3 per respondent – aim for >4 per respondent) |
| Deliverables | Anonymized PowerPoint charts |

ABBREVIATIONS USED:
CKD = chronic kidney disease
HD = haemodialysis
CKD-aP= chronic kidney disease-associated pruritus
UP = uremic pruritus (terminology used by nephrologists)

LIST OF TOPICS COVERED (not exhaustive):
Diagnosing UP: caseload, screening/reporting & classification
Treating UP: attitude, usage, image
Future of UP: expectations of UP market

ALL TEXT IN BLUE IS NOT SHOWN TO RESPONDENT, do not translate

AGENCY DETAILS:
19027005 CKD-aP Nephrologists
Cambridge Healthcare Research (Vox.Bio)

| Screener | | | | |
|---|---|---|---|---|
| S0. Quota | 1 | ○ | Germany | n=50 |
| | 2 | ○ | France | n=50 |
| | 3 | ○ | Italy | n=50 |
| | 4 | ○ | Spain | n=50 |
| | 5 | ○ | UK | n=50 |
| | 6 | ○ | Australia | n=50 |
| Intro | Thank you very much for agreeing to participate in this online survey. We are conducting a survey on the management and treatment of **hemodialysis patients** suffering from **CKD-associated pruritus**, sometimes also referred to as uremic pruritus.<br>On average, the survey will take approximately **60 minutes** to complete. The survey includes a general questionnaire (20 minutes), followed by 5 patient records (approx. 6–8 minutes each).<br>This study is being conducted by an independent market research agency (Vox.Bio) on behalf of a pharmaceutical company. The company who is commissioning this research does not hold or see any information regarding respondents. As a member of EphMRA and BHBIA, Vox.Bio is bound by the Code of Conduct and all applicable laws protecting your personal data and responses. The study is conducted in compliance with EphMRA and ESOMAR guidelines – the aim of this market research study is to gain your views and is not in any way promotional. The research will comply with GDPR. In the case of research conducted in the UK, we act in accordance with the ABPI, MRS and BHBIA codes of conduct.<br>With the exception of the note below regarding the recording and reporting of adverse events, other additional safety information, and/or product quality complaints, any information you disclose will be treated in the strictest confidence and the results are anonymized so no answers are attributable to any individuals.<br>You are about to enter a market research questionnaire. | | | |

| | |
|---|---|
| AE 1 | **Pharmacovigilance**<br>By law, we are required to pass on to our client details of adverse events, other additional safety information and/or product quality complaints that are mentioned during the course of market research interviews and surveys. Although this is an online market research questionnaire and how you respond will, of course, be treated in confidence, should you raise an adverse event, other additional safety information and/or product quality complaint in a specific patient or group of patients, we will need to report this even if it has already been reported by you directly to the company or to the regulatory authorities.<br>Progr: All countries except for Germany: In such a situation you will be contacted to ask whether or not you are willing to waive the confidentiality given to you under the Market Research Codes of Conduct specifically in relation to that adverse event, other additional safety information or product quality complaint. Everything else you contribute during the course of the questionnaire will continue to remain confidential.<br>Progr: Germany only: In such a situation you will remain anonymous and will be identified only by role (e.g. nurse, physician, pharmacist, patient, consumer). If further information is necessary, additional questions will be provided by a company via agency with retaining your confidentiality<br><br>**I confirm that I have been informed of the obligations for the collection and reporting of any adverse events, other additional safety information and/or product quality complaints related to the client's product that are noted by me during the course of this research.** |

| | | |
|---|---|---|
| 1 | I would like to proceed and protect my anonymity / I would like to proceed | |
| 2 | I would like to proceed and in case an adverse event is mentioned by me during this survey, I accept to be contacted by SERMO to discuss my feedback | |
| 3 | I don't want to proceed; end the interview here | Progr: Terminate |

| | |
|---|---|
| AE 2 | Progr: France only:<br>Concerning Market Research in France, according to French legislation Loi Bertrand and Loi Anti-Cadeaux, fieldwork agencies shall report all HCPs details partaking in this research on their centralized government website (https://www.entreprises-transparence.sante.gouv.fr & https://www.conseil-national.medecin.fr). |

| | | | |
|---|---|---|---|
| 1 | O | Yes, I agree | |
| 2 | O | No | Progr: Terminate |

| | |
|---|---|
| AE 3 | Progr: Germany and Italy only:<br>Please review the following statement: I confirm I do not need any consent from my employer, organization, or professional association to participate in this research OR I have already obtained any consent needed. |

| | | | |
|---|---|---|---|
| 1 | O | Yes, I confirm | |
| 2 | O | No, I do not confirm | Progr: Terminate |

| | |
|---|---|
| S1 | What is your primary **medical specialty?** |

| | | | |
|---|---|---|---|
| 1 | O | Nephrologist | |
| 99 | O | Other | Progr: Terminate |

| | |
|---|---|
| S2 | How many **years of experience** do you have in your specialty (after completion of formal training)? |

| | | | |
|---|---|---|---|
| 1 | O | Less than 3 years | Progr: Terminate |
| 2 | O | 3 to 10 years | |
| 3 | O | 11 to 20 years | |
| 4 | O | 21 to 30 years | |
| 5 | O | More than 30 years | Progr: Terminate |

| | |
|---|---|
| S3 | What proportion of your working time do you devote to **direct patient care** (i.e. excluding research and teaching)? |

| | | |
|---|---|---|
| 1 | ____ % of time devoted to direct patient care | Progr: Terminate if <70% (all except Spain) / <60% (Spain only) |

| | |
|---|---|
| S4 | In which **setting(s)** do you primarily practice? |

| | | | |
|---|---|---|---|
| 1 | O | Inpatient clinic (Klinik-Bereich) | Progr: Germany only |
| 2 | O | Outpatient clinic (Klinik-Ambulanz) | |
| 3 | O | Office (Praxis) & Dialysis center (Dialyse-Zentrum) | |
| 4 | O | Hospital | Progr: France only |
| 5 | O | Private center / clinic | |
| 6 | O | Association | |
| 7 | O | Hospital | Progr: Italy only |
| 8 | O | Private dialysis center | |
| 9 | O | Public hospital | Progr: Spain only |
| 14 | O | Private hospital | |
| 10 | O | Dialysis center | |
| 11 | O | Hospital | Progr: Auto-populate for UK |
| 12 | O | Hospital | Progr: Australia only |
| 13 | O | Satellite dialysis center | |

Progr: do not ask for UK, pre-populate

| | |
|---|---|
| S5 | How many **CKD patients** on the following types of dialysis did you personally treat **in the past one month**? And how many of these patients also suffer from **CKD-associated pruritus**? |

**By "CKD-associated pruritus", we mean <u>pruritus that persists between dialysis sessions</u> rather than an itch happening only during dialysis sessions**

| | | i. **Number of patients on dialysis** | ii. **Number of patients on dialysis with CKD-associated pruritus** |
|---|---|---|---|
| a) | Center / hospital-based hemodialysis | ____ patients<br>Range 0–999 | __ patients with CKD-associated pruritus<br>Terminate if <4<br>Progr: Value must be <S5ai<br>Range 0–S5a_i |
| b) | At-home peritoneal dialysis | ____ patients<br>Range 0–999 | __ patients with CKD-associated Pruritus<br>Progr: Value must be <S5bi<br>Range 0–S5b_i |

| | | | |
|---|---|---|---|
| c) | At-home hemodialysis | _____ patients<br>Range 0–999 | ___ patients with CKD-associated pruritus<br>Progr: Value must be <S5ci<br>Range 0–S5c_i |
| | **TOTAL** | Progr: Show running total<br>Terminate if sum <50 | Progr: Show running total |

**S6** Are you personally involved in making **treatment decisions** to manage **CKD-associated pruritus** in dialysis patients?

| | | | |
|---|---|---|---|
| 1 | O | Yes | |
| 2 | O | No | Progr: Terminate |

**S7** Within this study we will ask you to **document 5 hemodialysis patients with CKD-associated pruritus** who you have seen in consultation in the past 3 months. **We need you to kindly submit a minimum of 5 patient records of hemodialysis patients with CKD-associated pruritus**

The details we ask you to enter for each patient refer to individual characteristics such as age, time of diagnosis, comorbidities, details of dialysis, impact of CKD-associated pruritus on patient, current / previous treatment for CKD-associated pruritus.

Please confirm that you will be able to document 5 hemodialysis patients suffering from CKD-associated pruritus that you have seen.

| | | | |
|---|---|---|---|
| 1 | O | Yes, I confirm | |
| 2 | O | No, I cannot confirm | Progr: Terminate |

**S8** Do you currently work as a paid investigator, researcher or consultant for a pharmaceutical company (other than participating in clinical trials), advertising agency or marketing research company?

| | | | |
|---|---|---|---|
| 1 | O | Yes | Progr: Terminate |
| 2 | O | No | |

**S9** In **which region** is your hospital or practice located?
Progr: Display list per market from appendix
Progr: Soft quota – aim to recruit a geographical spread

Progr: Please collect full screening data from all respondents – i.e. each respondent should complete the screener in full and only terminate after S9

## Main questionnaire

Thank you very much for your responses so far. You have qualified for the research. The first part of this will be a questionnaire lasting approximately 20 minutes.

The questions will concern your general perception of CKD-associated pruritus and of the treatment options available to treat this condition.

## CKD-associated Pruritus Diagnostics

**Q1** Please review the following statements relating to **diagnosis of CKD-associated pruritus** and **indicate your level of agreement or disagreement with each**. Please answer on a scale of 1 to 7 where 1 means "Do not agree at all" and 7 means "Strongly agree".

*Please select one answer per row.*

| | Progr: Randomize rows | 1 – **Do not agree at all** | 2 | 3 | 4 | 5 | 6 | 7 – **Strongly agree** |
|---|---|---|---|---|---|---|---|---|
| a) | My institution / practice has a systematic approach to screening for CKD-associated pruritus | | | | | | | |

| | | | | | | | | |
|---|---|---|---|---|---|---|---|---|
| b) | CKD-associated pruritus is easy to diagnose using clinical observation alone | | | | | | | |
| c) | Diagnosis of CKD-associated pruritus is usually patient-driven (e.g. patient mentions itch symptoms) | | | | | | | |
| d) | There is a need to use a consistent international scale to diagnose CKD-associated pruritus | | | | | | | |
| e) | There is a need for new guidelines related to diagnosis of CKD-associated pruritus | | | | | | | |
| f) | CKD-associated pruritus is underdiagnosed within the hemodialysis patient population | | | | | | | |

**Q2A** When managing patients with CKD-associated pruritus, how do you **classify** your patients?

*Please select the answer(s) which most closely align with your classification*

| | | | |
|---|---|---|---|
| 1 | ☐ | Mild, Moderate, Severe | |
| 2 | ☐ | No itch / Itch | |
| 3 | ○ | Neither of the above [EXCLUSIVE] | |

Progr: Can code 1 &/OR 2

**Q2B** If Q2A=1: When classifying your CKD-associated pruritus patients as mild, moderate, severe, which of the following characteristics do you use in order to determine each degree of severity?

If Q2A=2 or 3: *If you were* to classify your CKD-associated pruritus patients as mild, moderate, severe, which of the following characteristics would you use in order to determine each degree of severity?

*Please tick all that apply for each column*

| | Characteristics | Mild CKD-associated pruritus | Moderate CKD-associated pruritus | Severe CKD-associated pruritus |
|---|---|---|---|---|
| 1) | Itch persists following dialysis optimization | ☐ | ☐ | ☐ |
| 2) | Complain of occasional itching | ☐ | ☐ | ☐ |
| 3) | Complain of continuous itching | ☐ | ☐ | ☐ |
| 4) | Complain of itch having negative impact on daily activities | ☐ | ☐ | ☐ |
| 12) | Complain of itch having negative impact on overall mood / mental health | ☐ | ☐ | ☐ |
| 5) | Has difficulties sleeping due to the itch | ☐ | ☐ | ☐ |
| 6) | Visible scratch marks due to itching | ☐ | ☐ | ☐ |
| 11) | Visible skin abrasions due to itching | ☐ | ☐ | ☐ |
| 7) | Itch persists despite treatment with topical moisturizers (e.g. Nivea) / emollients | ☐ | ☐ | ☐ |

| | | | | |
|---|---|---|---|---|
| 9) | Itch persists despite treatment with antihistamines | ☐ | ☐ | ☐ |
| 10) | Itch persists despite treatment with gabapentinoids (e.g. gabapentin / pregabalin) | ☐ | ☐ | ☐ |

Progr: Multiple answers allowed per row; must answer at least once per column.

**Q2C** Of the [PIPE IN S5a_ii] hemodialysis patients with CKD-associated pruritus you personally treated in the past month, how many fit the following classifications of CKD-associated pruritus you just described?

| | | |
|---|---|---|
| i) | **Mild CKD-associated pruritus** | _____ patients |
| ii) | **Moderate CKD-associated pruritus** | _____ patients |
| iii) | **Severe CKD-associated pruritus** | _____ patients |
| iv) | **Unknown severity of CKD-associated pruritus** | _____ patients |
| | **TOTAL** | Progr: Show running total<br>Progr: Sum must equal S5a_ii |

Progr: Range 0–999

**Q2D** What factors do you consider when deciding what treatment to provide to a patient with CKD-associated pruritus?

*Please provide as much detail as possible*

| |
|---|
| |

Progr: open verbatim

**Q2F** Do you use any **itch scales** to **determine severity** of a patient's CKD-associated pruritus?

| | | |
|---|---|---|
| 1 | ○ | Yes |
| 2 | ○ | No |

**Q2G** Progr: Show dynamically on same screen as Q2F if respondent answers Q2F=1

**Which itch scales** do you use to determine severity of a patient's CKD-associated pruritus?

| | Randomize rows (except Other specify)<br>Allow multiple responses | |
|---|---|---|
| 1) | 5-D itch scale | ☐ |
| 2) | 12-Item Pruritus Severity Score (12-PSS) | ☐ |
| 3) | Dermatology Quality of Life Index (DQLI) | ☐ |
| 4) | Dynamic Pruritus Score (DPS) | ☐ |
| 5) | Itchy-QoL | ☐ |
| 6) | Leuven Itch Scale (LIS) | ☐ |
| 7) | Numerical Rating Scale (NRS) / Visual Analogue Scale (VAS) | ☐ |
| 8) | Other, please specify ________ | ☐ |

Progr: Ask if Q2F=1

## Current Treatment Options

**Q3** Please select the response which best describes **your awareness and / or usage** with each of the following types of treatment / intervention for CKD-associated pruritus (CKD-aP).

| | |
|---|---|
| | *Please choose one answer per type of treatment.*<br>Progr: Columns:<br>a) Dialysis optimization<br>b) Topical moisturizers (e.g. Nivea) / Emollients<br>c) Topical antihistamines<br>d) Topical corticosteroids<br>e) Oral antihistamines<br>f) Oral corticosteroids<br>g) Gabapentinoids (e.g. gabapentin / pregabalin)<br>h) Antidepressants / Anxiolytics / Sedatives<br>i) Opioid receptor modulators<br>j) UVB therapy<br>m) Other, please specify ________ [Progr, allow only selections in codes 2 to 6 for this option]<br>Progr: Rows:<br>1. I **have not heard** of this as a treatment for CKD-aP<br>2. I **have heard** of this as a treatment for CKD-aP, but have **only limited experience** with it<br>3. I am **very knowledgeable** about this as a treatment for CKD-aP, but have **only limited experience** with it<br>4. I regard this treatment as one of my **standard therapies for a select group** of CKD-aP patients<br>5. I regard this treatment as one of my **standard therapies for a broad population** of CKD-aP patients<br>6. I regard this treatment as **my therapy of choice** [EXCLUSIVE]<br>7. I **do not use / consider** this option<br>Progr: Columns. Randomize except "Dialysis optimization" (anchor as first column) and "UVB therapy" (anchor as last column). Retain same order in subsequent treatment list questions. One answer per column (Grid)<br>Programmer: Show error message if all treatments are selected with Code 1 AND/OR 7: "You have stated that you have not heard or do not use / consider any of the options. Please review your answers before continuing." Force a change.<br>Programmer: Show error message if all treatments are selected with Code 1 AND/OR 7: "You have stated that you have not heard or do not use / consider any of the options. Please review your answers before continuing." Force a change.<br>Progr: option 6 Exclusive and should only be chosen once |
| Q4 | You mentioned you treat your CKD-associated pruritus patients with the following treatment types / interventions. **Which severity level(s) of CKD-associated pruritus** do you typically prescribe each treatment type / intervention for?<br><br>If you do not typically classify your patients into mild, moderate, severe, please answer based on the characteristics you selected earlier regarding each degree of severity.<br><br>*Please select all that apply* |

| | **Treatments / Interventions**<br>Progr: Hide treatments where rows 1 or 7 selected at Q3<br>Same randomization as in Q3 | **Mild CKD-associated pruritus** | **Moderate CKD-associated pruritus** | **Severe CKD-associated pruritus** |
|---|---|---|---|---|
| 1) | Dialysis optimization | ☐ | ☐ | ☐ |
| 2) | Topical moisturizers (e.g. Nivea) / Emollients | ☐ | ☐ | ☐ |
| 4) | Topical antihistamines | ☐ | ☐ | ☐ |
| 5) | Topical corticosteroids | ☐ | ☐ | ☐ |

| | | | | | |
|---|---|---|---|---|---|
| | 6) | Oral antihistamines | ☐ | ☐ | ☐ |
| | 7) | Oral corticosteroids | ☐ | ☐ | ☐ |
| | 8) | Gabapentinoids (e.g. gabapentin / pregabalin) | ☐ | ☐ | ☐ |
| | 15) | Antidepressants / Anxiolytics / Sedatives | ☐ | ☐ | ☐ |
| | 12) | Opioid receptor modulators | ☐ | ☐ | ☐ |
| | 10) | UVB therapy | ☐ | ☐ | ☐ |
| | 11) | [PIPE IN OE RESPONSE FROM Q3k] | ☐ | ☐ | ☐ |

Progr: Skip if Q2A=1

Multiple answers allowed per row; must answer at least once per row

**Q5A**

A) What proportion of your CKD-associated pruritus patients currently receive a combination of treatments?

| | |
|---|---|
| 1 | ______ % of patients receiving a combination of treatments |

Progr: Range = 0–100%

**Q5b**

Thinking of all your patients with CKD-associated pruritus, what percentage, if any, do you refer to each of the following physician types (for treatment of their CKD-aP)?

If you do not refer any of your patients to a particular physician type, please leave the relevant cell blank.

| | Randomize rows (except Other specify)<br>Allow multiple responses | % of patients referred |
|---|---|---|
| 1) | Dermatologist | ____% |
| 2) | General practitioner | ____% |
| 3) | Palliative care specialist | ____% |
| 4) | Internal medicine specialist | ____% |
| 5) | Allergist / Immunologist | ____% |
| 6) | Other, please specify ________ | ____% |

Progr: Range for each cell = 0–100%
Progr: Autofill empty cells with 0

**Q5c**

Progr: Ask if Q5b_1 (Derm) >0%

For what reason(s) do you refer your CKD-aP patients to a dermatologist?

| | Allow multiple responses | |
|---|---|---|
| 1) | Overall management of CKD-aP | ☐ |
| 2) | UVB therapy | ☐ |
| 3) | Other, please specify ________ | ☐ |

**Q6**

For each of the following lines of therapy, please indicate your ***typical* treatment approach** for CKD-associated pruritus. This may be a monotherapy or combination approach. If you use a combination of treatment types at a particular line of therapy, please select all of these together.

You may include a specific treatment type at more than one line of therapy, provided it is part of a different combination from the previous line of therapy.

Please provide answers for at least 3 lines of therapy. If you treat patients with 4 or more lines of therapy, you are welcome to continue selecting treatment regimens.

Progr: ASK THIS QUESTION IF Q2a CODE 2 OR 3 SELECTED ONLY. SKIP IF Code 1 SELECTED AT Q2a

| | **Treatments / Interventions**<br>Progr: Hide treatments where rows 1 or 7 selected at Q3<br><br>Prog, show order based on responses to Q3. Code 6 (therapy of choice) at top down to code 2 (have heard of but limited experience) at bottom. If >1 treatment given same rating at Q3, fine to sort alphabetically / at random | **1st line of therapy** | **2nd line of therapy** | **3rd line of therapy** | **4th line of therapy** | **5th line of therapy** | **6th line of therapy** |
|---|---|---|---|---|---|---|---|
| 1) | Dialysis optimization | ☐ | ☐ | ☐ | ☐ | ☐ | ☐ |
| 2) | Topical moisturizers (e.g. Nivea) / Emollients | ☐ | ☐ | ☐ | ☐ | ☐ | ☐ |
| 4) | Topical antihistamines | ☐ | ☐ | ☐ | ☐ | ☐ | ☐ |
| 5) | Topical corticosteroids | ☐ | ☐ | ☐ | ☐ | ☐ | ☐ |
| 6) | Oral antihistamines | ☐ | ☐ | ☐ | ☐ | ☐ | ☐ |
| 7) | Oral corticosteroids | ☐ | ☐ | ☐ | ☐ | ☐ | ☐ |
| 8) | Gabapentinoids (e.g. gabapentin / pregabalin) | ☐ | ☐ | ☐ | ☐ | ☐ | ☐ |
| 15) | Antidepressants / Anxiolytics / Sedatives | ☐ | ☐ | ☐ | ☐ | ☐ | ☐ |
| 12 | Opioid receptor modulators | ☐ | ☐ | ☐ | ☐ | ☐ | ☐ |
| 10) | UVB therapy | ☐ | ☐ | ☐ | ☐ | ☐ | ☐ |
| 11) | [PIPE IN OE RESPONSE FROM Q3k] | ☐ | ☐ | ☐ | ☐ | ☐ | ☐ |

PROG: change this question to a drag and drop card sorter to ensure each therapy line is considered distinctly. New line of therapy to self-populate with previous therapies to be kept or removed.

Drag and drop any of the treatment options to create ‘1st line of therapy’. Once they have submitted this, the heading should show ‘2nd line of therapy’ – and the treatment options they have just selected at 1st line should be shown within the bucket. But they will then need to add / remove treatment options to create their ‘2nd line of therapy’. This process will be repeated for 3rd line, 4th line etc. – each time, pre-populating the bucket with the response from the line immediately prior

Prog, Once 4th line of therapy appears, also display a new card – 'I do not treat patients at this line of therapy’. Option should be exclusive and only selectable at 4th, 5th and 6th line
Progr: Do not allow identical selection in 2 consecutive columns (e.g. answer for 3rd-line therapy cannot be identical to answer for 2nd-line therapy); enforce minimum of 3 lines of therapy

| Q6b | For each of the following lines of therapy, please indicate your ***typical* treatment approach** for CKD-associated pruritus *by severity classification*. This may be a monotherapy or combination approach. If you use a combination of treatment types at a particular line of therapy, please select all of these together in the same column.<br><br>You may include a specific treatment type at more than one line of therapy, provided it is part of a different combination from the previous line of therapy.<br><br>Please provide answers for at least 3 lines of therapy. If you treat patients with 4 or more lines of therapy, you are welcome to continue selecting treatment regimens.<br><br>ASK THIS QUESTION ONLY IF Q2a CODE 1 SELECTED. SKIP IF Code 1 NOT SELECTED AT Q2a<br><br>REPEAT THE QUESTION 3 TIMES, FOR MILD, MODERATE AND SEVERE |
|---|---|

| | | Q6b [Show different header according to the corresponding loop: **[Mild / Moderate / Severe] CKD-associated pruritus** | | | | | |
|---|---|---|---|---|---|---|---|
| | **Treatments / Interventions**<br>Progr: Hide treatments where rows 1 or 7 selected at Q3 [DELETED-Same randomization as in Q3]<br><br>Prog, show order based on responses to Q3. Code 6 (therapy of choice) at top down to code 2 (have heard of but limited experience) at bottom. If >1 treatment given same rating at Q3, fine to sort alphabetically / at random | **1st line of therapy** | **2nd line of therapy** | **3rd line of therapy** | **4th line of therapy** | **5th line of therapy** | **6th line of therapy** |
| 1) | Dialysis optimization | ☐ | ☐ | ☐ | ☐ | ☐ | ☐ |
| 2) | Topical moisturizers (e.g. Nivea) / emollients | ☐ | ☐ | ☐ | ☐ | ☐ | ☐ |
| 4) | Topical antihistamines | ☐ | ☐ | ☐ | ☐ | ☐ | ☐ |
| 5) | Topical corticosteroids | ☐ | ☐ | ☐ | ☐ | ☐ | ☐ |
| 6) | Oral antihistamines | ☐ | ☐ | ☐ | ☐ | ☐ | ☐ |
| 7) | Oral corticosteroids | ☐ | ☐ | ☐ | ☐ | ☐ | ☐ |
| 8) | Gabapentinoids (e.g. gabapentin / pregabalin) | ☐ | ☐ | ☐ | ☐ | ☐ | ☐ |
| 15) | Antidepressants / Anxiolytics / Sedatives | ☐ | ☐ | ☐ | ☐ | ☐ | ☐ |
| 12 | Opioid receptor modulators | ☐ | ☐ | ☐ | ☐ | ☐ | ☐ |
| 10) | UVB therapy | ☐ | ☐ | ☐ | ☐ | ☐ | ☐ |
| 12) | [PIPE IN OE RESPONSE FROM Q3k] | ☐ | ☐ | ☐ | ☐ | ☐ | ☐ |

Prog: Use same drag and drop format as Q6

Prog. Once 4th line of therapy appears, also display a new card – 'I do not treat patients at this line of therapy'. Option should be exclusive and only selectable at 4th, 5th and 6th line

| | |
|---|---|
| | Progr: Do not allow identical selection in 2 consecutive columns (e.g. answer for 3rd-line therapy cannot be identical to answer for 2nd-line therapy); enforce minimum of 3 lines of therapy |
| Q8 | Below are several criteria which may or may not be important when selecting a treatment for patients suffering from CKD-associated pruritus.<br><br>On a scale of 1 to 7 where 1 means "Not important at all" and 7 means "Extremely important", please **rate how important you believe each criteria to be** when making treatment decisions for patients with **CKD-associated pruritus**. |

| | **Features of treatment** Progr: Randomize rows within categories | 1 – **Not important at all** | 2 | 3 | 4 | 5 | 6 | 7 – **Extremely important** |
|---|---|---|---|---|---|---|---|---|
| **Treatment properties** | | | | | | | | |
| a) | Reduces severity of itch | | | | | | | |
| b) | Rapid onset of action | | | | | | | |
| c) | Sustained duration of response | | | | | | | |
| d) | Good efficacy in monotherapy | | | | | | | |
| e) | Unique mechanism of action | | | | | | | |
| f) | Low side-effect profile | | | | | | | |
| g) | Good tolerability | | | | | | | |
| **Route of administration** | | | | | | | | |
| h) | Convenient mode of administration (e.g. oral) | | | | | | | |
| i) | Convenient dosing frequency | | | | | | | |
| **Patients' quality of life** | | | | | | | | |
| j) | Helps to overcome emotional impact of CKD-associated pruritus (e.g. anxiety, depression, overall mood, self-consciousness) | | | | | | | |
| k) | Helps to improve day-to-day functioning (e.g. social, occupational) | | | | | | | |
| **Additional criteria** | | | | | | | | |
| n) | Cost-effective | | | | | | | |
| o) | Previous positive personal experience | | | | | | | |
| p) | High likelihood of patient compliance | | | | | | | |

| | |
|---|---|
| Q9 | Using a scale of 1 to 7 where 1 means "Extremely dissatisfied" and 7 means "Extremely satisfied", please **rate how satisfied you are with the performance of the following treatments** in patients with **CKD-associated pruritus**.<br><br>*Please select one answer per row.*<br><br>Progr: Repeat grid for following therapies:<br><br>1. **Topical / Oral antihistamines** (Show If Q3d and/or Q3f=2-6)<br>2. **Topical / Oral corticosteroids** (Show If Q3e and/or Q3g=2-6) |

3. **Gabapentinoids (e.g. gabapentin / pregabalin)** (Show If Q3h=2-6)
4. **Antidepressants / Anxiolytics / Sedatives** (Show If Q3j=2-6)
6. **Opioid receptor modulators** (Show If Q3k=2-6)
5. **UVB therapy** (Show If Q3m=2-6)

| | **Features of treatment** PROG: Randomize rows b–e | 1 – **Extremely dissatisfied** | 2 | 3 | 4 | 5 | 6 | 7 – **Extremely satisfied** |
|---|---|---|---|---|---|---|---|---|
| a) | Overall satisfaction with treatment | | | | | | | |
| b) | Efficacy in monotherapy | | | | | | | |
| c) | Tolerability | | | | | | | |
| d) | Convenient mode of administration [Progr: Show for treatments 3–6 only] | | | | | | | |
| e) | Impact on patient's quality of life | | | | | | | |

## Attitudes towards treating CKD-associated pruritus

**Q10** Please review the following statements relating to **treatment / management of CKD-associated pruritus** and **indicate your level of agreement or disagreement with each**. Please answer on a scale of 1 to 7 where 1 means "Do not agree at all" and 7 means "Strongly agree".

*Please select one answer per row.*

| | Progr: Randomize rows | 1 – **Do not agree at all** | 2 | 3 | 4 | 5 | 6 | 7 – **Strongly agree** |
|---|---|---|---|---|---|---|---|---|
| a) | For patients with bothersome CKD-associated pruritus, treatment options are very limited | | | | | | | |
| b) | CKD-associated pruritus represents a minor concern to physicians when considering the broader context of a patient's CKD | | | | | | | |
| c) | There is a need for new treatments specifically designed to address CKD-associated pruritus | | | | | | | |
| e) | There is a need for new guidelines related to treating CKD-associated pruritus | | | | | | | |
| f) | When a patient first presents with CKD-associated pruritus, I already have a clear plan of treatment in my mind | | | | | | | |
| g) | Compared to other conditions, I am more willing to try new therapies when treating bothersome CKD-associated pruritus | | | | | | | |

| | | | | | | | | |
|---|---|---|---|---|---|---|---|---|
| h) | It is important that patients with bothersome CKD-associated pruritus are treated with drug therapy (for their CKD-aP) | | | | | | | |
| j) | Dialysis optimization can cure CKD-associated pruritus in most patients | | | | | | | |
| k) | CKD-associated pruritus should be treated by a dermatologist | | | | | | | |

**Q11** We would now like to understand your expectations for the future CKD-associated pruritus market.

a) Firstly, please **enter which treatment(s) you consider to be the standard of care** for CKD-associated pruritus?

| |
|---|
| |

Progr: open verbatim

b) Now, for each of the following attributes, compared to the current standard of care, **what level of improvement do you feel is *needed*** when thinking about future treatments for CKD-associated pruritus?

*Please select one answer per row.*

Progr: Columns.

a) Minor / No improvement over current standard of care needed
b) Moderate improvement over current standard of care needed
c) Major improvement over current standard of care needed

Progr: Rows. Randomize.

1. Effect on itch
2. Onset of action
3. Durability of response
4. Efficacy in monotherapy
5. Novel mechanisms of action
6. Side-effect profile
7. Convenience of mode of administration
8. Convenience of dosing frequency
9. Ability to overcome emotional impact of CKD-associated pruritus
10. Ability to improve day-to-day function of patients with CKD-associated pruritus
11. Ability to improve patient quality of life in general
12. Cost-effectiveness
13. Easy for patients to comply with

**Q12** What treatments / products, if any, are you aware of that are **currently being developed or have recently been approved** for the treatment of **CKD-associated pruritus**?

| |
|---|
| |
| |
| |

Progr: OE. Show 3 boxes – force minimum of 3 characters per box
Progr: Also include a mutually exclusive checkbox: I am not aware of any treatments that are currently being developed or have recently been approved for the treatment of CKD-associated pruritus
Progr: Must enter answer into at least 1 OE box OR select checkbox

Thank & Close

| Patient Record Forms | |
|---|---|
| | Each respondent is asked to provide 5 PRFs covering CKD patients:<br>• seen in consultation in the last 3 months AND<br>• currently receiving hemodialysis AND<br>• with CKD-associated pruritus AND<br>• is currently or has previously received treatment for their CKD-associated pruritus<br><br>See form at the end of this document<br><br>Programmer: Show each group of questions under the same table header in one screen. I.e, “Patient characteristics” :, questions 1 to 3 in the same screen, and so on.<br><br>Thank you very much for your responses so far – you have completed the questionnaire and will now move onto the patient record forms.<br><br>We would like you to **document 5 hemodialysis patients with CKD-associated pruritus** who you have seen in consultation in the past 3 months. These patients must be currently receiving or have previously received treatment / an intervention for their CKD-associated pruritus. We anticipate this will take approximately 40 minutes.<br><br>If possible, please try to tell us about patients with **differing levels of CKD-associated pruritus severity.** |

[Prog, add subsequent number of forms 1/5, 2/5, 3/5, 4/5, 5/5 at the top of the screen]

# PATIENT RECORD FORM: Patient with CKD-associated pruritus AND currently receiving hemodialysis (HD) AND seen in consultation in the last 3 months.

**Important: Please aim to cover a range of CKD-associated pruritus severity levels/ experience across your PRFs**

## Patient characteristics

**1.** Gender
1○ Male
2○ Female

**2.** Current Age
|__|__| years Range 0–99

**3.** Current Body weight
|__|__|__| kg Range 0–200

## Last consultation

**4.** Date of last consultation:
|__|__||__|__||__|__|__|__|
dd mm yyyy

Min date allowed: 01/12/2919
Prog, if date introduced is earlier than 3 months ago, show the following error message "The consultation date you have entered is more than 3 months ago. The patients you are recording should have been seen in consultation within the last 3 months. Please review your response."

**5a.** Current P/PTH level

|__|__|

Range 0.0–999.9
Prog: Show a drop-down next to the numeric box with the following units for the respondent to chose: "pg/mL" OR "pmol/L"

**5b.** Current Ca level
|__|__|

Range 0.0–999.9
Prog: Show a drop-down next to the numeric box with the following units for the respondent to chose: "mg/dL" OR "mmol/L"

**5c.** Current CRP level

|__|__|

Range 0.0–999.9
Prog: Show a drop-down next to the numeric box with the following units for the respondent to chose: "mg/L" OR "mmol/L"

**5d.** Current Kt/V level

|__|__||
Range 0.0–9.9

## Symptomology of CKD-associated Pruritus

**6a.** Date when CKD-associated pruritus started:

1○ Within last 6 months
2○ 6 months to 1 year ago
3○ 1 year to 2 years ago
4○ 2 to 3 years ago
5○ More than 3 years ago

☐Not known

**7.** Perceived current severity of CKD-associated pruritus (PRFs should cover patients with a range of current severities)
1○ Mild
2○ Moderate
3○ Severe
10○ Not known / Not documented
Single code

**8.** Anatomical locations of CKD-associated pruritus
1☐ Face / Head
2☐ Neck
3☐ Arms
4☐ Hands
5☐ Back
6☐ Torso / Abdomen
7☐ Legs
8☐ Feet
9☐ Other, please specify: ________
10○ Not known / Not documented
Multiple answers, except code 10

**9.** Current reported impact of CKD-associated pruritus on patient quality of life
*Answer on a scale of 1 to 7 where 1 is 'No impact at all' and 7 is 'Significant impact'*

| | 1 | 2 | 3 | 4 | 5 | 6 | 7 | Don't know |
|---|---|---|---|---|---|---|---|---|
| Day-to-day functioning | ○ | ○ | ○ | ○ | ○ | ○ | ○ | ○ |
| Sleep quality | ○ | ○ | ○ | ○ | ○ | ○ | ○ | ○ |
| Aesthetics of skin | ○ | ○ | ○ | ○ | ○ | ○ | ○ | ○ |
| Emotional wellbeing | ○ | ○ | ○ | ○ | ○ | ○ | ○ | ○ |
| Relationships | ○ | ○ | ○ | ○ | ○ | ○ | ○ | ○ |

One answer per row

## Hemodialysis

**11.** Location of hemodialysis (currently)
1○ Hospital / Dialysis center
2○ At home
Single code

**12a.** Time since started dialysis

Please indicate in years / months how long ago the patient started receiving dialysis
|__|__| *(months)* |__|__| *(years)*

Prog: Range for months 0-11. Range for years 0–50
Progr: Respondent not allowed to enter '0' for both Years and Months. Auto-fill with 0 if only one box is answered.

☐Not known

**12b.** Modality of dialysis (currently)
|__|__| times per week Range 0–9
for
|__|__|__| hours per session Range 0.0–9.9 [Allow one decimal]

**12c.** Type of hemodialysis
1○ Conventional hemodialysis (HD)
2○ Hemodiafiltration (HDF)
Single code

| | | | |
|---|---|---|---|
| | | **10.** **Dermatological complications of CKD-associated pruritus**<br>1☐ Impetigo<br>2☐ Prurigo<br>3☐ Chronic, lichenified dermatitis / eczema<br>4○ None<br>5○ Not documented / Don't know<br>**Multiple answers; Codes 4–5 cannot be combined with other codes** | |

| 1st treatment for CKD-associated pruritus | 2nd treatment for CKD-associated pruritus | 3rd treatment for CKD-associated pruritus |
|---|---|---|

13. Initial / 1st treatment for CKD-associated pruritus
Please select the type(s) of treatment which represented your **first line approach** to this patient. If the patient is on a combination treatment, please select each treatment option/part of the combination

**Progr. Hide all options selected with codes 1 or 7 in Q3 (Main Part) Show as card sort drag and drop**
1☐ Dialysis optimization
2☐ Topical moisturizers / Emollients
4☐ Topical antihistamines
5☐ Topical corticosteroids
6☐ Oral antihistamines
7☐ Oral corticosteroids
8☐ Gabapentinoids
14☐ Antidepressants / Anxiolytics / Sedatives
10☐ Opioid receptor modulators
12☐ UVB phototherapy
98☐ Other treatment for CKD-associated pruritus.
**Multiple answer for codes 1–98**

**Progr: If options 2–11 or 14 or 98 selected, display OE box**
You indicated the patient received drug therapy. Please write in details of the product(s) used (e.g. name of product / molecule).

**14. Length of time on this treatment**

For how long did the patient receive this treatment / combination of treatment? Please provide your answer in years / months. For reference, you indicated this patient's CKD-associated pruritus started [PIPE IN 6a]

|__|__| *(months)* |__|__| *(years)*
**Range for months 0–11. Range for years 0–50 Allow 1 decimal**
**Progr: Respondent not allowed to enter '0' for both Years and Months. Auto-fill with 0 if only one box is answered.**

☐Not known

**15. Key reason for prescribing this treatment**

What was the key reason for prescribing this treatment to this patient?

**16. Overall, how satisfied were you with this treatment?**
*Answer on a scale of 1 to 7 where 1 is 'Not at all satisfied' and 7 is 'Extremely satisfied'*

**18. After the initial treatment approach that you indicated, how did this patient's treatment regimen change for CKD-associated pruritus?**
The box below has been populated with the initial / 1st treatment the patient received, please **update this box to reflect the next regimen they received**. You may do this by adding and/or removing treatments.

**Progr. Hide all options selected with codes 1 or 7 in Q3 (Main Part) Show as card sort drag and drop pre-populate based on Q13**

1☐ Dialysis optimization
2☐ Topical moisturizers / Emollients
4☐ Topical antihistamines
5☐ Topical corticosteroids
6☐ Oral antihistamines
7☐ Oral corticosteroids
8☐ Gabapentinoids
14☐ Antidepressants / Anxiolytics / Sedatives
10☐ Opioid receptor modulators
12☐ UVB phototherapy
98☐ Other treatment for CKD-associated pruritus.
○ Not currently receiving treatment for CKD-associated pruritus → **End PRF.**

**Multiple answers**

**Progr: If options 2–11 or 14 or 98 selected, display OE box**
For any new or switch drug therapies the patient was receiving, please also write in details of the product(s) used (e.g. name of product / molecule).

**19. Length of time on this treatment**

For how long did the patient receive this treatment / combination of treatment? Please provide your answer in years / months. For reference, you indicated this patient's CKD-associated pruritus started [PIPE IN 6a]

|__|__| *(months)* |__|__| *(years)*
**Range for months 0-11. Range for years 0–50 Allow 1 decimal**
**Progr: Respondent not allowed to enter '0' for both Years and Months. Auto-fill with 0 if only one box is answered.**

☐Not known

**20. Reason for treatment switch / add-on**

What was the reason for this treatment switch / add-on in this patient?

**23. After the treatment approaches you have highlighted, how has this patient's treatment regimen changed for CKD-associated pruritus?**
The box below has been populated with the patient's previous treatment regimen, please **update this box to reflect the next regimen they received**. You may do this by adding and/or removing treatments.

**Progr. Hide all options selected with codes 1 or 7 in Q3 (Main Part) Show as card sort drag and drop pre-populate based on Q18**

1☐ Dialysis optimization
2☐ Topical moisturizers / Emollients
4☐ Topical antihistamines
5☐ Topical corticosteroids
6☐ Oral antihistamines
7☐ Oral corticosteroids
8☐ Gabapentinoids
14☐ Antidepressants / Anxiolytics / Sedatives
10☐ Opioid receptor modulators
12☐ UVB phototherapy
98☐ Other treatment for CKD-associated pruritus.
○ Not currently receiving treatment for CKD-associated pruritus → **End PRF.**

**Multiple answer**

**Progr: If options 2–11 or 14 or 98 selected, display OE box**
For any new or switch drug therapies the patient was receiving, please also write in details of the product(s) used (e.g. name of product / molecule).

**24. Length of time on this treatment**

For how long did the patient receive this treatment / combination of treatment? Please provide your answer in years / months. For reference, you indicated this patient's CKD-associated pruritus started [PIPE IN 6a]

|__|__| *(months)* |__|__| *(years)*
**Range for months 0–11. Range for years 0–50 Allow 1 decimal**
**Progr: Respondent not allowed to enter '0' for both Years and Months. Auto-fill with 0 if only one box is answered.**

☐Not known

**25. Reason for treatment switch / add-on**

What was the reason for this treatment switch / add-on in this patient?

| | 1 | 2 | 3 | 4 | 5 | 6 | 7 |
|---|---|---|---|---|---|---|---|
| Overall satisfaction with treatment | O | O | O | O | O | O | O |

**17. Is this the only treatment the patient has received for CKD-associated pruritus?**

1O Yes → End PRF
2O No → Go to Q18

**21. Overall, how satisfied were you with this treatment?**
*Answer on a scale of 1 to 7 where 1 is 'Not at all satisfied' and 7 is 'Extremely satisfied'*

| | 1 | 2 | 3 | 4 | 5 | 6 | 7 |
|---|---|---|---|---|---|---|---|
| Overall satisfaction with treatment | O | O | O | O | O | O | O |

**22. Is this the patient's current treatment for CKD-associated pruritus?**
1O Yes → End PRF
2O No → Go to Q23

**26. Overall, how satisfied were you with this treatment?**
*Answer on a scale of 1 to 7 where 1 is 'Not at all satisfied' and 7 is 'Extremely satisfied'*

| | 1 | 2 | 3 | 4 | 5 | 6 | 7 |
|---|---|---|---|---|---|---|---|
| Overall satisfaction with treatment | O | O | O | O | O | O | O |

**27. Is this the patient's current treatment for CKD-associated pruritus?**
1O Yes → End PRF
2O No → Go to Q28 – i.e. next loop (see note below)

**Progr: Continue loop of questioning until respondent answers 'Yes' to the question 'Is this the current treatment…'. Up to a maximum of 3 more loops (i.e. capturing a maximum of 6 treatments, including 'current treatment'). Loop refers to Q23 - Q27. So, if resp answers 'No' at Q27, they should do another loop of these questions (23-27). Up to a maximum of 3 loops.**

**Replace header with:**
**4th treatment for CKD-associated pruritus**
**5th treatment for CKD-associated pruritus**
**6th treatment for CKD-associated pruritus**

## Appendix: List of regions for use at S9

| France | Germany | Italy | Spain | UK | Australia |
| --- | --- | --- | --- | --- | --- |

| | | | | | |
|---|---|---|---|---|---|
| 1. Auvergne Rhône-Alpes<br>2. Bourgogne Franche-Comté<br>3. Bretagne<br>4. Centre Val de Loire<br>5. Corse<br>6. Grand Est<br>7. Hauts-de-France<br>8. Île-de-France<br>9. Normandie<br>10. Nouvelle-Aquitaine<br>11. Occitanie<br>12. Pays de la Loire<br>13. Provence-Alpes-Côte d'Azur | 1. Baden-Württemberg<br>2. Berlin<br>3. Brandenburg<br>4. Freie Hansestadt Bremen<br>5. Freie und Hansestadt Hamburg<br>6. Freistaat Bayern<br>7. Freistaat Sachsen<br>8. Freistaat Thüringen<br>9. Hessen<br>10. Mecklenburg-Vorpommern<br>11. Niedersachsen<br>12. Nordrhein-Westfalen<br>13. Rheinland-Pfalz<br>14. Saarland<br>15. Sachsen-Anhalt<br>16. Schleswig-Holstein | 1. Abruzzo<br>2. Basilicata<br>3. Calabria<br>4. Campania<br>5. Emilia-Romagna<br>6. Friuli-Venezia Giulia<br>7. Latium<br>8. Liguria<br>9. Lombardia<br>10. Marche<br>11. Molise<br>12. Piemonte<br>13. Puglia<br>14. Sardegna<br>15. Sicilia<br>16. Toscana<br>17. Trentino-Alto Adige<br>18. Umbria<br>19. Valle d'Aosta<br>20. Veneto | 1. Andalucía<br>2. Aragón<br>3. Asturias<br>4. Baleares<br>5. Canarias<br>6. Cantabria<br>7. Castilla y León<br>8. Castilla la Mancha<br>9. Cataluña<br>10. Comunidad Valenciana<br>11. Extremadura<br>12. Galicia<br>13. La Rioja<br>14. Madrid<br>15. Murcia<br>16. Navarra<br>17. País Vasco | 1. East Midlands<br>2. Greater London<br>3. North East England<br>4. North West England<br>5. South East England<br>6. South West England<br>7. West Midlands<br>8. Yorkshire and the Humber<br>9. Scotland<br>10. Wales<br>11. Northern Ireland | 1. Australian Capital Territory<br>2. New South Wales<br>3. Northern Territory<br>4. Queensland<br>5. South Australia<br>6. Tasmania<br>7. Victoria<br>8. Western Australia |
